# Regorafenib Alteration of the BCL-xL/MCL-1 Ratio Provides a Therapeutic Opportunity for BH3-Mimetics in Hepatocellular Carcinoma Models

**DOI:** 10.3390/cancers12020332

**Published:** 2020-02-01

**Authors:** Blanca Cucarull, Anna Tutusaus, Miguel Subías, Milica Stefanovic, Tania Hernáez-Alsina, Loreto Boix, María Reig, Pablo García de Frutos, Montserrat Marí, Anna Colell, Jordi Bruix, Albert Morales

**Affiliations:** 1 Department of Cell Death and Proliferation, IIBB-CSIC, IDIBAPS, 08036 Barcelona, Spain; Blanca.cucarull@iibb.csic.es (B.C.); anna.tutusaus@iibb.csic.es (A.T.); msubias97@hotmail.com (M.S.); ms.milica.stefanovic@gmail.com (M.S.); pablo.garcia@iibb.csic.es (P.G.d.F.); monmari@clinic.cat (M.M.); anna.colell@iibb.csic.es (A.C.); 2 Departament de Biomedicina, Facultat de Medicina, Universitat de Barcelona, 08036 Barcelona, Spain; 3 Department of Radiation Oncology, Catalan Institute of Oncology (ICO)-IDIBELL, L’Hospitalet, 08908 Barcelona, Spain; 4 Digestive Unit, Hospital San Pedro, Rioja Salud, 26006 La Rioja, Spain; taniahernaez@gmail.com; 5 Barcelona Clinic Liver Cancer Group, Liver Unit, Hospital Clínic of Barcelona, University of Barcelona, CIBEREHD, IDIBAPS, 08036 Barcelona, Spain; LBOIX@clinic.cat (L.B.); MREIG1@clinic.cat (M.R.); JBRUIX@clinic.cat (J.B.); 6 Centro de Investigación Biomédica en Red sobre Enfermedades Cardiovasculares (CIBERCV), Spain; 7 Centro de Investigación Biomédica en Red sobre Enfermedades Neurodegenerativas (CIBERNED), Spain

**Keywords:** liver cancer, mitochondria, apoptosis, Bcl-2 family, A-1331852, combination therapy

## Abstract

Background: The multikinase inhibitor regorafenib, approved as second-line treatment for hepatocellular carcinoma (HCC) after sorafenib failure, may induce mitochondrial damage. BH3-mimetics, inhibitors of specific BCL-2 proteins, are valuable drugs in cancer therapy to amplify mitochondrial-dependent cell death. Methods: In in vitro and in vivo HCC models, we tested regorafenib’s effect on the BCL-2 network and the efficacy of BH3-mimetics on HCC treatment. Results: In hepatoma cell lines and Hep3B liver spheroids, regorafenib cytotoxicity was potentiated by BCL-xL siRNA transfection or pharmacological inhibition (A-1331852), while BCL-2 antagonism had no effect. Mitochondrial outer membrane permeabilization, cytochrome c release, and caspase-3 activation mediated A-1331852/regorafenib-induced cell death. In a patient-derived xenograft (PDX) HCC model, BCL-xL inhibition stimulated regorafenib activity, drastically decreasing tumor growth. Moreover, regorafenib-resistant HepG2 cells displayed increased BCL-xL and reduced MCL-1 expression, while A-1331852 reinstated regorafenib efficacy in vitro and in a xenograft mouse model. Interestingly, BCL-xL levels, associated with poor prognosis in liver and colorectal cancer, and the BCL-xL/MCL-1 ratio were detected as being increased in HCC patients. Conclusion: Regorafenib primes tumor cells to BH3-mimetic-induced cell death, allowing BCL-xL inhibition with A-1331852 or other strategies based on BCL-xL degradation to enhance regorafenib efficacy, offering a novel approach for HCC treatment, particularly for tumors with an elevated BCL-xL/MCL-1 ratio.

## 1. Introduction

Hepatocellular carcinoma (HCC), the most frequent primary liver cancer, is the third leading cause of cancer death and the main cause of death among patients with cirrhosis [1]. Often diagnosed at an advanced stage with poor prognosis, its incidence is expected to rise in the future due to the growing prevalence of non-alcoholic fatty liver disease associated with obesity and metabolic syndrome [2]. Despite recent advances in treatment, HCC prognosis continues to be dismal [3]. Most liver cancer patients do not benefit from immunotherapy [4] and the efficacy of the multikinase inhibitors (MKIs) sorafenib [5] and lenvatinib [6] in first-line treatment, and regorafenib [7] and cabozantinib [8] in second line, needs to be improved. Since drug effectiveness is limited by primary and acquired drug resistance [9], the identification of mechanisms enhanced by chemotherapy, particularly those susceptible to being druggable, is required to overcome treatment failure. In HCC, with a complex genetic background and without dependence on specific driver mutations for survival, vulnerabilities created by MKI treatment could provide targets to improve life expectancy [10].

Cell death-related pathways involving mitochondria are gaining interest as an alternative approach for cancer therapy [11], especially after or in combination with drug treatment that has altered mitochondrial homeostasis [10]. The BCL-2 network controls apoptosis by regulating mitochondrial outer membrane permeabilization (MOMP) via multidomain pro-apoptotic BAX and BAK [12]. MOMP triggers the release of pro-apoptotic mitochondrial intermembrane space proteins, such as cytochrome c and smac/DIABLO, activating executioner caspases and rapid cell death. In the BCL-2 system, equilibrium is established among pro-apoptotic members, such as BID, BIM, PUMA, BAD, or NOXA, and pro-survival components, mainly BCL-2, BCL-xL, and MCL-1 [13]. Cancer therapy has been described to alter the delicate balance established between activators and repressors of BAX/BAK homo-oligomerization, favoring the MOMP and leading to cell death. Upon the appearance of drug resistance, compensatory mechanisms may cause a novel BCL-2 status, which could be profited by BH3-mimetics [14,15,16], selective BCL-2 family member inhibitors studied in on-going clinical trials [17]. In particular, we and others have demonstrated sorafenib interaction with mitochondria [18,19,20,21], indicating the BCL-2 system has an important role in its cytotoxicity, which could be used to increase sorafenib efficacy [22,23]. Regorafenib shares a chemical structure and biological targets with sorafenib [24,25] and BCL-2 seems to participate in death-signaling pathways induced by both drugs [23]. Knowing the BCL-2 profile induced by a drug helps design a strategy based on BH3-mimetics predicted to be successful for a specific cancer [26,27,28]. However, unlike sorafenib, regorafenib’s effect on the BCL-2 network has not been sufficiently addressed, so we aimed to evaluate this point and to test potential combination therapies in different models of liver cancer. 

Our results indicate that MCL-1 reduction, as regorafenib does, allows BCL-xL antagonism to effectively eliminate HCC cells. In fact, A-1331852, a BH3-mimetic with specific anti-BCL-xL-binding capacity [29], is an effective agent to increase regorafenib efficacy and to overcome regorafenib resistance as we will demonstrate in different in vitro and in vivo HCC models. Moreover, increased BCL-xL and the BCL-xL/MCL-1 ratio are exhibited by patients with HCC, with predicted worse prognosis, suggesting that A-1331852 could be an interesting drug to combine with regorafenib during therapy.

## 2. Results

### 2.1. Mitochondrial Differences in Sorafenib vs. Regorafenib Experimental Liver Cancer Treatment

Sorafenib and regorafenib share numerous signaling pathways in their biological action, although some proteins are specifically targeted. Previous works have identified part of the cytotoxicity associated with sorafenib as mitochondrial dependent, with sorafenib activity being potentiated by mitochondrial-directed therapies. Differences in sorafenib- and regorafenib-induced pathways could provide additional targets for combination therapy and identify a mechanism that leads liver cancer cells to death.

**Figure 1:**
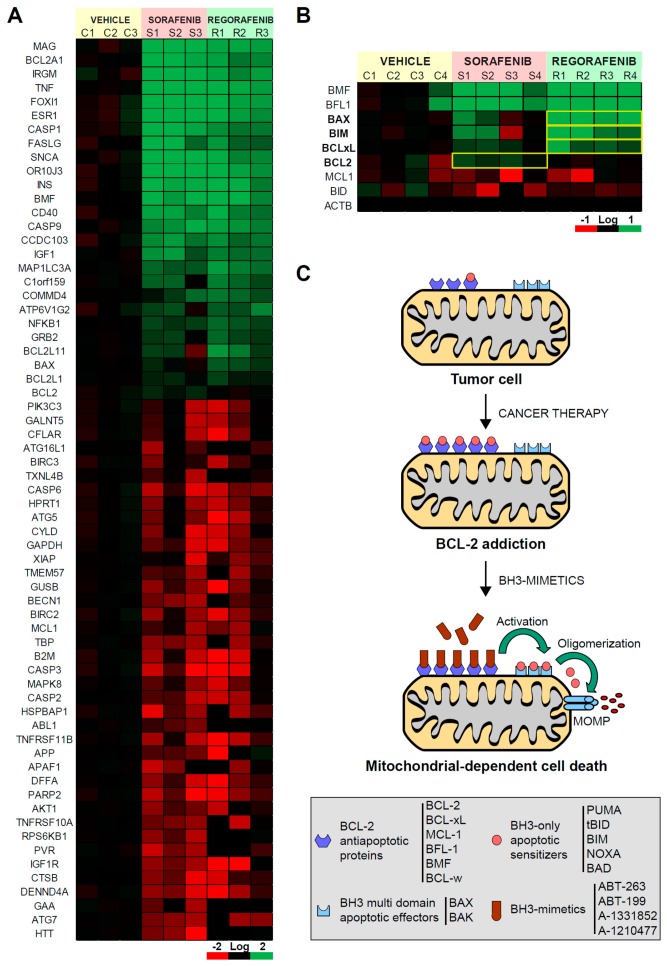
Sorafenib and regorafenib regulate the BCL-2 profile differently, sharing mitochondrial dependence but a distinctive therapeutic approach. (**A**) Transcriptomic analysis of genes related with liver cancer in BCLC9 tumors from nude mice treated for three weeks with vehicle (C1-3), sorafenib (S1-3), or regorafenib (R1-3). (**B**) mRNA expression of different BCL-2 proteins from treated tumors (C1-4, S1-4, and R1-4). Differences in the mRNA pattern are highlighted with yellow squares. (**C**) Cancer therapy may increase anti-apoptotic BCL-2 proteins avoiding cell death but mito-priming the cells to BH3-mimetics. Resistant hepatoma cells treated with compounds targeting BCL-2 proteins may release BH3-only proteins to bind BAX/BAK and trigger apoptotic cell death.

In a patient-derived xenograft mouse model, we evaluated the effect of sorafenib and regorafenib in HCC using a microarray with a panel of cell death-related genes (Figure 1A). Although most of the mRNAs detected were similarly affected by both MKIs, changes in individual genes were detected. In particular, we observed that the alteration in BCL-2 family members was clearly different in sorafenib- and regorafenib-treated tumors. Anti-apoptotic members, such as BMF or BFL1, were mostly upregulated after both treatments. However, while the BCL-2 increase was mainly noticed after sorafenib exposure, BCL-xL, augmented more significantly in regorafenib-treated tumors (Figure 1B). Of note, the expression of pro-apoptotic members, such as BIM or BAX, was more pronounced after regorafenib treatment. These results evidenced different alterations in BCL-2 proteins induced by both MKIs, suggesting divergent mitochondrial effects and specific therapeutic opportunities for each. 

Several chemotherapeutic agents disturb the mitochondrial BCL-2 network, increasing both pro-apoptotic and pro-survival BCL-2 family members (Figure 1C). As a result, in surviving cancer cells, drug therapy generates an abnormal BCL-2 balance with high levels of opposite components on each scale. This equilibrium is breakable by specific BH3-mimetics, which lead to cell death after sequestering the anti-apoptotic BCL-2 members. Since regorafenib upregulates BCL-2 expression, particularly pro-apoptotic genes, such as BIM and BAX, the priming of mitochondrial cell death should be expected. Therefore, we decided to investigate if BCL-2 addiction is created by regorafenib exposure and which proteins could be targeted to increase regorafenib efficacy.

### 2.2. BCL-xL Antagonism Is Effective to Potentiate Regorafenib Activity Against Liver Cancer Cells

**Figure 2:**
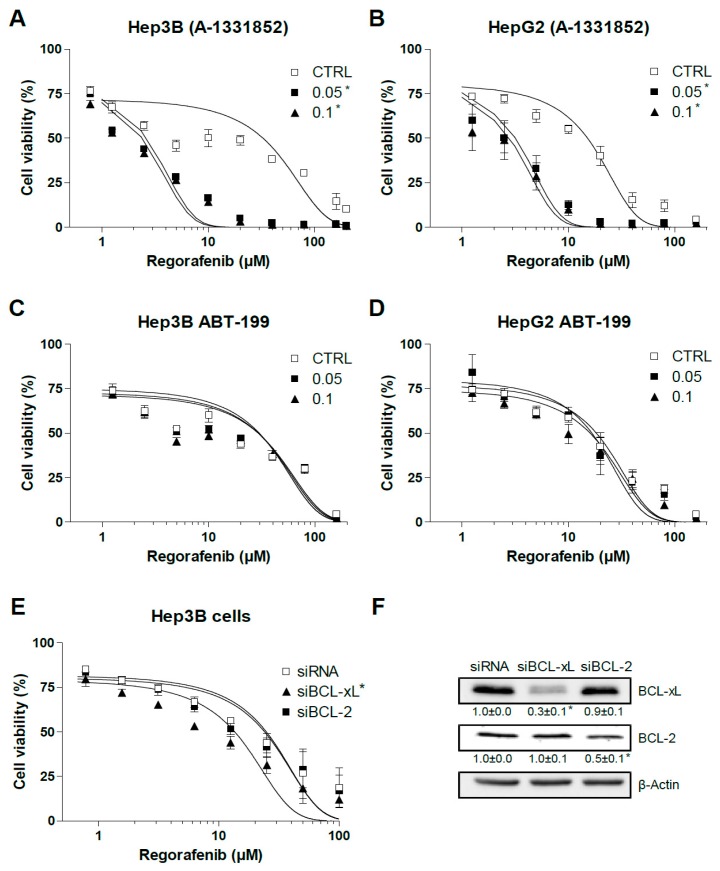
BCL-xL antagonism potentiates regorafenib activity on liver cancer cells. (**A**,**B**) Hep3B and HepG2 cells were treated for 16 h with the BCL-xL inhibitor A-1331852 and regorafenib at different concentrations, and cell viability was quantified by MTT. (**C**,**D**) Hep3B and HepG2 cells were treated for 16 h with the BCL-2 inhibitor ABT-199 and regorafenib at different concentrations, and cell viability was quantified by MTT. (**E**) Hep3B cells were transfected with siRNA control or against BCL-xL and BCL-2 and after 48 h treated with regorafenib at different concentrations, and cell viability was quantified by MTT. (**F**) RNA interference was confirmed and protein levels of BCL-xL, BCL-2, and β-actin are shown in parallel panels. (n = 3) * *p* < 0.05 vs. control or siCTRL cells.

We observed previously that BCL-2 and BCL-xL are the main anti-apoptotic BCL-2 proteins involved in sorafenib resistance in hepatoma liver cancer cells [23]. Since the BH3-mimetics ABT-199 [30] and A-1331852 [29] are highly effective to specifically reduce the intracellular availability of BCL-2 and BCL-xL, respectively, we tested if these compounds could modify regorafenib activity. A-1331852 greatly potentiated regorafenib toxicity in Hep3B and HepG2 cells as measured in MTT assays after 16 h (Figure 2A,B), while BCL-2 depletion with ABT-199 was not effective in increasing regorafenib action in the same hepatoma cell lines (Figure 2C,D). Of note, addition of the anti-BCL-xL BH3-mimetic A-1331852 significantly increased regorafenib-induced cell death, up to 5–6 fold (EC50: 15.1 ± 1.3 vs. 2.8 ± 0.3) in HepG2 cells and 8 to 10 times (30.7 ± 4.3 vs. 2.4 ± 0.2) in Hep3B cells. 

To verify BCL-xL’s role in the cellular protection against regorafenib, we transfected siBCL-2 and siBCL-xL in Hep3B cells (Figure 2E). Cells transfected with siBCL-2 were not sensitized against regorafenib while BCL-xL silencing potentiated cell death after 24 h of regorafenib exposure (EC50: 24.8 ± 3.5 vs. 13.6 ± 1.9). Of note, the A-1331852 efficacy of sensitizing tumor cells against regorafenib was higher than siBCL-xL reduction, probably due to A-1331852’s powerful inhibition (Ki < 0.04 nM) of BCL-xL compared with the reduction obtained, up to 80% (Figure 2F), with the two siBCL-xL tested. However, in the absence of total knockdown of BCL-xL, we cannot completely discard the contribution of some off-target effect on the increased regorafenib efficacy.

**Figure 3:**
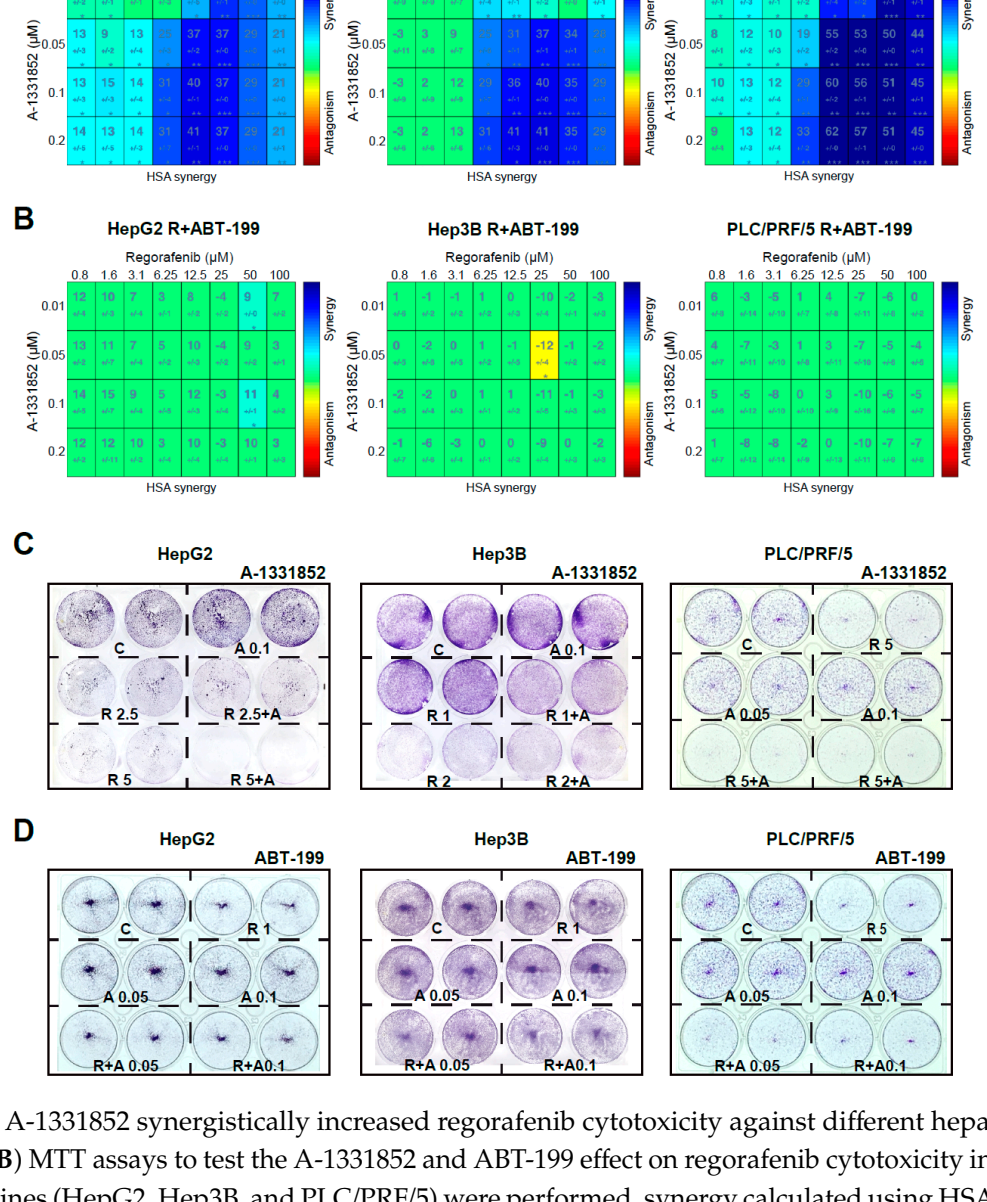
A-1331852 synergistically increased regorafenib cytotoxicity against different hepatoma cell lines. (**A**,**B**) MTT assays to test the A-1331852 and ABT-199 effect on regorafenib cytotoxicity in different liver cell lines (HepG2, Hep3B, and PLC/PRF/5) were performed, synergy calculated using HSA analysis, and results displayed with heat maps (blue synergy vs. red antagonism). (**C**,**D**), Crystal Violet staining was performed after 3 days of treatment with vehicle (**C**), regorafenib (R), and/or A-1331852/ABT-199 (**A**) in HepG2, Hep3B, and PLC/PRF/5 cell cultures. (n = 3) * *p* < 0.05 vs. control.

To validate the capacity of A-1331852 to potentiate regorafenib toxicity, we evaluated their potential synergism in three different liver cancer cell lines, using the mathematic Highest Single Agent (HSA) model [31] and presenting heat maps of the results (Figure 3A). Synergy between both agents, regorafenib and A-1331852, was clearly observed in all three hepatoma cells, HepG2, Hep3B, and PLC/PRF/5, for concentrations of BH3-mimetic in the nanomolar range (10–200 nM) at a regorafenib concentration with therapeutic relevance in the low micromolar range (1–100 µM). 

In contrast, no synergism was detected when BCL-2 was the protein targeted using ABT-199 co-administration with regorafenib in any cell line tested (Figure 3B).

In agreement with these results, the growth of HepG2, Hep3B, and PLC/PRF/5 cells was severely decreased by the combination of A-1331852 and regorafenib after three days, as denoted by Crystal Violet assays (Figure 3C). In contrast, ABT-199 was ineffective, potentiating regorafenib activity over all three hepatoma cell lines (Figure 3D). This result suggests that BCL-xL antagonism, but not BCL-2, could be an interesting mechanism to increase regorafenib efficacy in vivo.

### 2.3. A-1331852 Addition to Regorafenib-Treated Hepatoma Cells Triggers MMP Loss and Mitochondrial-Mediated Caspase-Dependent Apoptotic Cell Death

**Figure 4:**
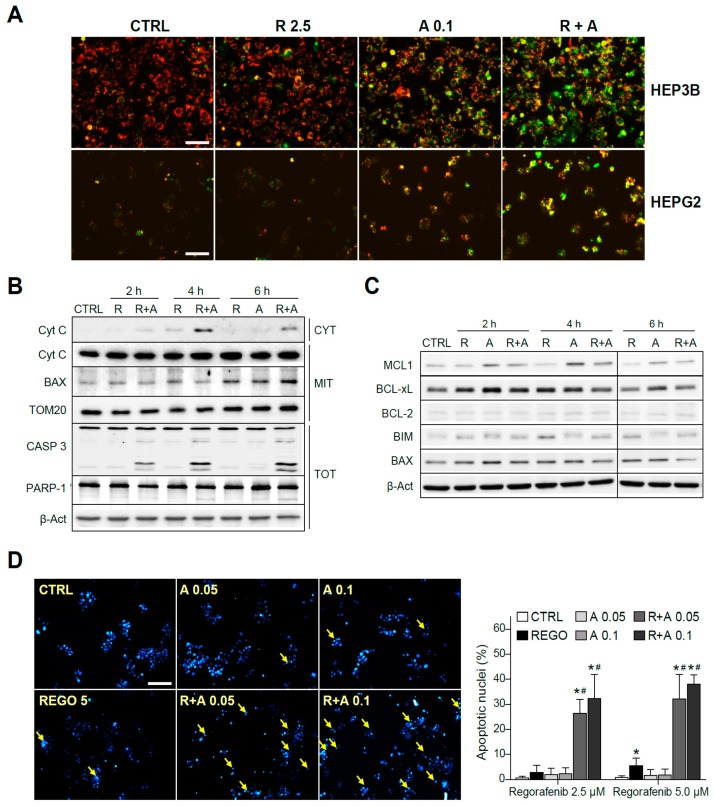
The regorafenib and A-1331852 combination induced apoptotic cell death via a mitochondrial caspase-dependent mechanism. (**A**) Hep3B and HepG2 cells were exposed to regorafenib (R, 2.5 μM) with or without A-1331852 (**A**, 0.1 µM) and MMP loss observed by fluorescence microscopy after 3 h (scale bar, 100 µm). (**B**) Cytochrome c release, BAX and TOM20 mitochondrial levels, caspase-3, PARP-1, and β-Actin were analyzed by Western blot in HepG2 cells. (**C**) BCL-2 proteins in cell extracts as above. (**D**) Nuclear Hoechst 33258 staining was visualized in HepG2 cells treated with regorafenib and/or A-1331852 (scale bar, 100 µm), and apoptotic cells counted (10 independent fields per condition, n = 3). * *p* < 0.05 vs. control cells, # *p* < 0.05 vs. regorafenib-treated cells.

To verify the mitochondrial alteration induced by A-1331852 in regorafenib-treated cells, we analyzed possible changes in the mitochondrial membrane potential (MMP) by using the fluorescence probe JC-1. As soon as three hours after the drugs’ co-administration, an evident decrease of the MMP was observed, denoted by the color shift observed in the cells, increasing the green mitochondrial pattern mainly in A-1331852/regorafenib-treated HepG2 and Hep3B cells (Figure 4A). 

Since the decline of MMP could be associated to mitochondrial pore formation and consequent release of mitochondrial pro-apoptotic intermembrane proteins, we measured the cytosolic levels of cytochrome c at different times. As detected by Western blot, while regorafenib alone induced a minimal amount of cytochrome c presence in the cytosol (CYT), A-1331852 co-administration greatly favored its mitochondrial release (Figure 4B). In mitochondrial extracts (MITs), whereas cytochrome c levels were significantly unchanged in the combination samples, BAX exhibited mitochondrial accumulation after regorafenib treatment. Consistent with a mitochondrial-dependent apoptotic cell death, a significant increase in the active caspase-3 form is clearly visible in regorafenib-treated cells only if A-1331852 was co-administered (Figure 4B). This result was confirmed by quantification of the caspase-3 activity in cell extracts (Figure S1). As a consequence of caspase-3 activation, a cleavage of PARP-1 was detectable in A-1331852/regorafenib-treated HepG2 cells (Figure 4B). 

To further analyze the early changes in BCL-2 proteins before caspase-3 triggering of cell death, we evaluated their protein levels. Once again, MCL-1 was clearly decreased in regorafenib-treated cells that was followed by BIM increases (Figure 4C). Other changes in BCL-2 proteins were not so clear, particularly due to their alteration in the levels induced by the BH3-mimetic A-1331852. Of note, while BAX mitochondrial accumulation was evident after regorafenib treatment, the BAX increase in total cell extracts was barely noticeable, emphasizing the importance of their mitochondrial analysis.

Moreover, typical apoptotic features were observed in hepatoma cells, being easily detectable by Hoechst 33258 nuclear staining after eight hours of A-1331852/regorafenib co-administration (Figure 4D). Interestingly, as previously observed with caspase-3 activation, nuclear DNA condensation was significant at time points where regorafenib alone was not inducing evident apoptotic effects, supporting a quick and relevant role of BCL-xL to modulate regorafenib anti-tumoral activity. 

### 2.4. Regorafenib Reduction of MCL-1 Facilitates A-1331852 Induction of Cell Death in Liver Cancer Cells

**Figure 5:**
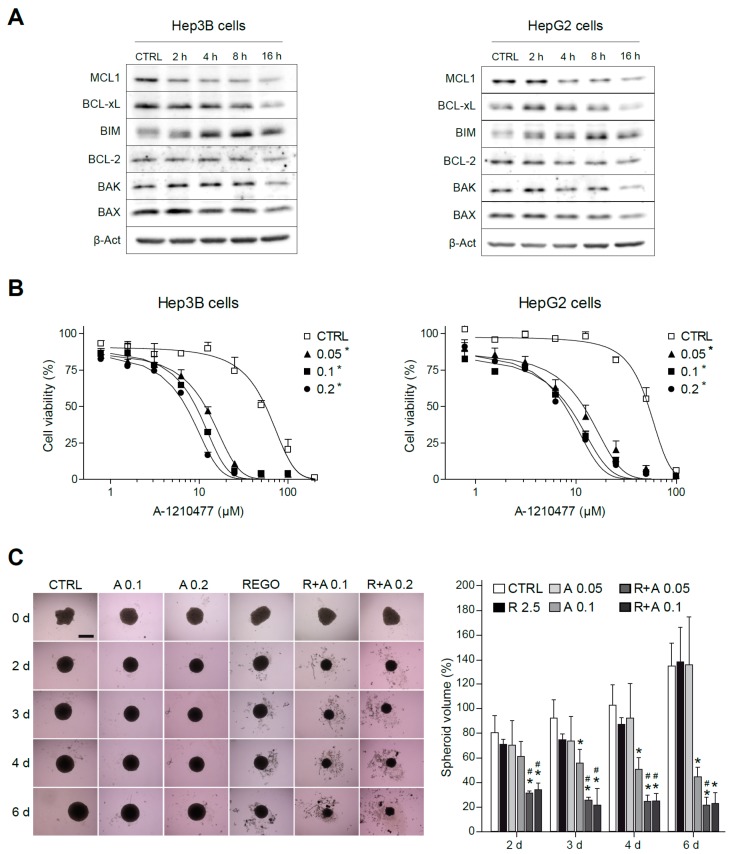
MCL-1 inhibition sensitizes hepatoma cells to the BCL-xL inhibitor A-1331852. (**A**) Representative Western blot images of MCL-1, BCL-xL, BIM, BCL-2, BAX, BAK, and β-Actin exhibited by Hep3B and HepG2 cells at different times (0–16 h) after regorafenib treatment (5 µM). (**B**) Effect of the MCL-1 inhibitor A-1210477 on Hep3B cells and HepG2 cells treated with A-1331852 (**A**, 0.05, 0.1, or 0.2 µM) for 24 h. * *p* < 0.05 vs. control cells. (**C**) Hep3B spheroids were seeded and after 24 h of aggregation treated with vehicle, regorafenib (R, 2.5 μM), and/or A-1331852 (A, 0.1 or 0.2 µM) for seven days. Spheroid growth was monitored daily (scale bar, 500 µm). (n = 3) * *p* < 0.05 vs. control cells, # *p* < 0.05 vs. regorafenib-treated cells.

To better identify the mitochondrial changes induced by regorafenib that allow BCL-xL antagonism to synergistically induce cytotoxicity in hepatoma cells, we analyzed the protein levels of BCL-2 members with recognized importance in cell survival. In Hep3B and HepG2 cells treated with regorafenib, an early decrease in MCL-1 levels was consistently observed, accompanied by a progressive increase in intracellular BIM levels (Figure 5A). Of note, this MCL-1 reduction was not caused by decreased mRNA synthesis. After overnight treatment with concentrations up to 5 µM of regorafenib, no significant decreases in MCL-1 mRNA were detected (Figure S2). Besides transcriptional modulation, MCL-1 expression is also tightly controlled by post-transcriptional modification [32,33], suggesting that proteasomal degradation of MCL-1 could be taking place in regorafenib-treated hepatoma cells. Although other mitochondrial alterations were detected, such as an increase in BCL-xL, particularly in regorafenib-treated HepG2 cells, MCL-1 reduction was presented by all cell lines tested after regorafenib treatment. 

Since a novel BH3-mimetic, A-1210477 [34], highly specific for MCL-1 has been recently described, we tested it, in order to deplete MCL-1 levels in hepatoma cells and combined with BCL-xL reduction using A-1331852. Interestingly, MCL-1 sequestration by A-1210477 was sufficient to induce BCL-xL-dependent cell death in liver cancer cell lines, such as HepG2 and Hep3B (Figure 5B). Therefore, this result suggests that the quick MCL-1 protein decline induced by regorafenib may be responsible for the BCL-xL addiction created in regorafenib-treated hepatoma cells, revealing a vulnerability that allows A-1331852 to be an effective anti-tumoral agent.

Before starting animal studies, we validated our results in liver spheroids, as a physiologically relevant in vitro HCC model, which resembles human liver more closely than traditional monolayer cultures. After aggregation, Hep3B spheroids were treated with regorafenib and/or A-1331852 and grown for seven days (Figure 5C). As quantified, two days after treatment, the drug combination was already effective in reducing spheroid growth while regorafenib activity was clearly minor and anti-BCL-xL-treatment alone was not significantly different from vehicle-treated spheroids.

### 2.5. A-1331852 in Combination with Regorafenib Is Effective to Reduce Liver Cancer Progression in a PDX Mouse Model

**Figure 6:**
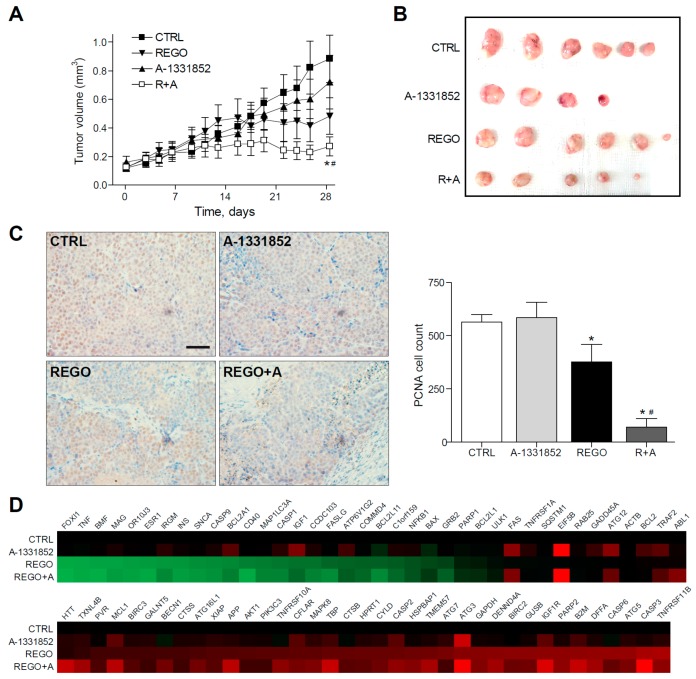
BCL-xL inhibitor A-1331852 remarkably reduced tumor growth in regorafenib-treated PDXs. (**A**,**B**), Subcutaneous growth quantification and images of BCLC9 tumors in mice treated with A-1331852 (25 mg/kg) and regorafenib (30 mg/kg) for 4 weeks (n = 4–6). * *p* < 0.05 vs. vehicle-treated mice. (**C**) Representative images of PCNA expression in tumor samples from BCLC9 PDXs and quantification (scale bar, 50 µm). (**D**) Transcriptomic analysis of cell death-related genes in BCLC9 tumors from nude mice treated with vehicle, regorafenib, and/or A-1331852. (n = 2).

To test the in vivo efficacy of BCL-xL antagonism to potentiate regorafenib activity against liver cancer, we administered regorafenib and A-1331852 to mice bearing BCLC9 tumors, generated after the subcutaneous injection of this patient-derived HCC cell line. BCLC9 are anchor-free growing human hepatocellular carcinoma cells, derived from a well-differentiated human HCC, that display a stem cell phenotype and are highly effective tumor-initiating cells in nude mice. Regorafenib’s capacity to decrease BCLC9 tumor growth was potentiated by A-1331852 co-administration (Figure 6A,B) while A-1331852 alone did not influence cancer progression significantly. In agreement, the proliferative capacity of the HCC cells was seriously compromised after regorafenib/A-1331852 co-administration for four weeks, as denoted by PCNA staining of tumor biopsies (Figure 5C). 

Quantification of PCNA-positive cells by field exhibited a regorafenib reduction of tumor proliferation (377 ± 140), compared to vehicle-treated animals (563 ± 62) that was potently increased by A-1331852 co-administration (70 ± 73). In contrast, A-1331852 in monotherapy was not observed to decrease tumor development (586 ± 141). Moreover, the presence of the tumor marker Ki-67 was confirmed in the BCLC9 xenografts (Figure S3). 

Of note, no changes in transaminase levels were induced by A-1331852, suggesting no hepatocellular damage induction by the BH3-mimetic to non-tumorous tissue. No major toxicity of A-1331852 was found in primary mouse hepatocytes (Figure S4) and in the human hepatic stellate cell line LX2 (Figure S5) at working concentrations, with cytotoxicity concentrations 50% (CC50s) more than 100-fold higher. Since regorafenib efficacy was clearly increased by BCL-xL inhibition in our PDX model, we evaluated the changes in regorafenib signaling introduced upon the A-1331852 combination, using a commercial microarray for cell death-related genes (Figure 6D). Interestingly, we found not only changes in BCL-2 family members, such as BFL1, BCL-xL, or BAX, but also downregulation in other genes. For instance, ATG12 and ATG3, which regulate mitochondrial homeostasis and autophagy in cell death [35], or IGF1 and IGF1R, were increased in HCC and proposed as targets for therapy [36], or the translation initiation factor EIF5B is modified by A-1331852 administration. These observations suggest an A-1331852 mitochondrial effect but also in other pathways relevant in HCC treatment. 

### 2.6. Regorafenib Resistant Cells Are Sensitive to A-1331852 Co-Administration in Vitro and In Vivo

**Figure 7:**
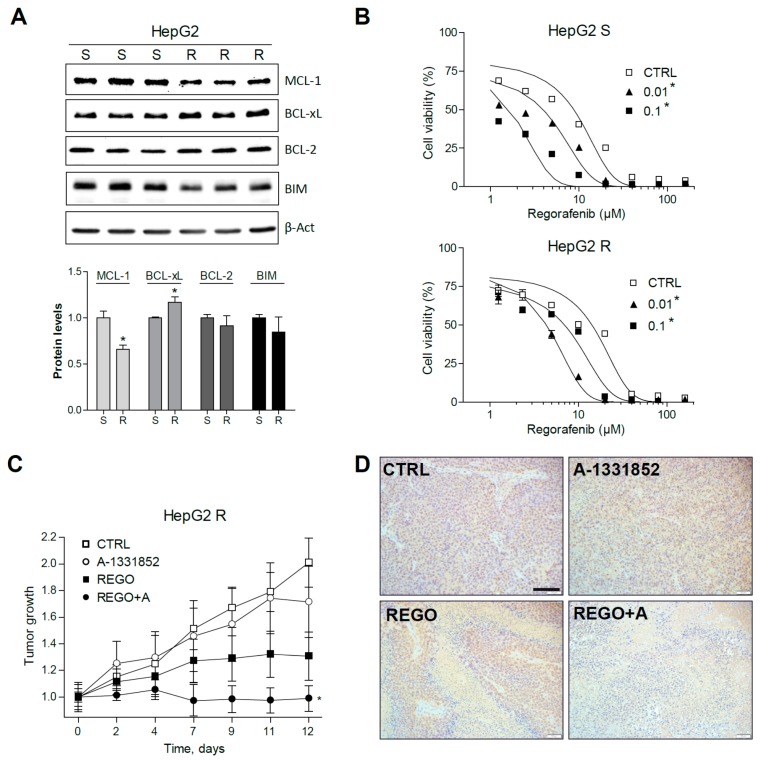
Regorafenib-resistant HepG2 cells, exhibiting mRNA changes in BCL-xL and MCL-1, are re-sensitized to regorafenib by A-1331582. (**A**) Representative Western blot images of MCL-1, BCL-xL, BCL-2, BIM, and β-Actin protein levels in S and R HepG2 cells. * *p* < 0.05 vs. sensitive cells. (**B**) Effect of A-1331852 (A, 0.01 or 0.1 µM) on S and R HepG2 cells. * *p* < 0.05 vs. control cells. (**C**) Subcutaneous growth of R HepG2 cells in mice treated orally with A-1331852 (25 mg/kg) and regorafenib (30 mg/kg) for 2 weeks (n = 4). (**D**) Representative images of PCNA expression in tumors from HepG2 R CDXs (scale bar, 100 µm). * *p* < 0.05 vs. vehicle-treated mice.

To know the protective mechanisms induced by regorafenib in resistant HCC tumors, a HepG2 cell line with regorafenib resistance was generated after 12 months of culture with regorafenib in the medium. An important MCL-1 reduction was accompanied by a significant BCL-xL increase in HepG2-resistant cells (R) compared to sensitive HepG2 cells (S), grown in parallel (Figure 7A). 

Interestingly, BCL-xL reduction partially abrogated regorafenib resistance (Figure 7B). For instance, regorafenib cytotoxicity against HepG2 R cells (EC50: 14.2 ± 1.8 µM) was increased by A-1331852 at nanomolar concentrations (EC50: 4.5 ± 0.4 µM, at 100 nM), even lower than the activity of regorafenib alone in sensitive cells (HepG2 S EC50: 8.7 ± 1.2 µM). 

To verify that A-1331852 was also effective in increasing regorafenib efficacy against regorafenib-resistant liver cancer cells in vivo, HepG2 R cells were injected subcutaneously in nude mice. As previously observed in the PDX BCLC9 model, regorafenib anti-tumoral activity was potentiated by A-1331852 administration in the HepG2 R xenograft model (Figure 7C). Accordingly, the quantification of PCNA-positive cells in the corresponding slides (Figure 7D) indicated that tumor proliferation was strongly diminished by regorafenib/A-1331852 co-administration (100 ± 88), compared to regorafenib- or vehicle-treated mice (869 ± 320 and 1573 ± 395, respectively). Moreover, to visualize cell death in the liver tumor specimens, TUNEL staining was performed. While no significant changes in TUNEL-positive cells were observed in A-1331852- and regorafenib-treated R HEPG2 tumors, mice receiving the combination treatment exhibited increased cell death (Figure S6).

### 2.7. BCL-xL Upregulation and MCL-1 Reduction Are Present in HCC Tumor Tissue

**Figure 8:**
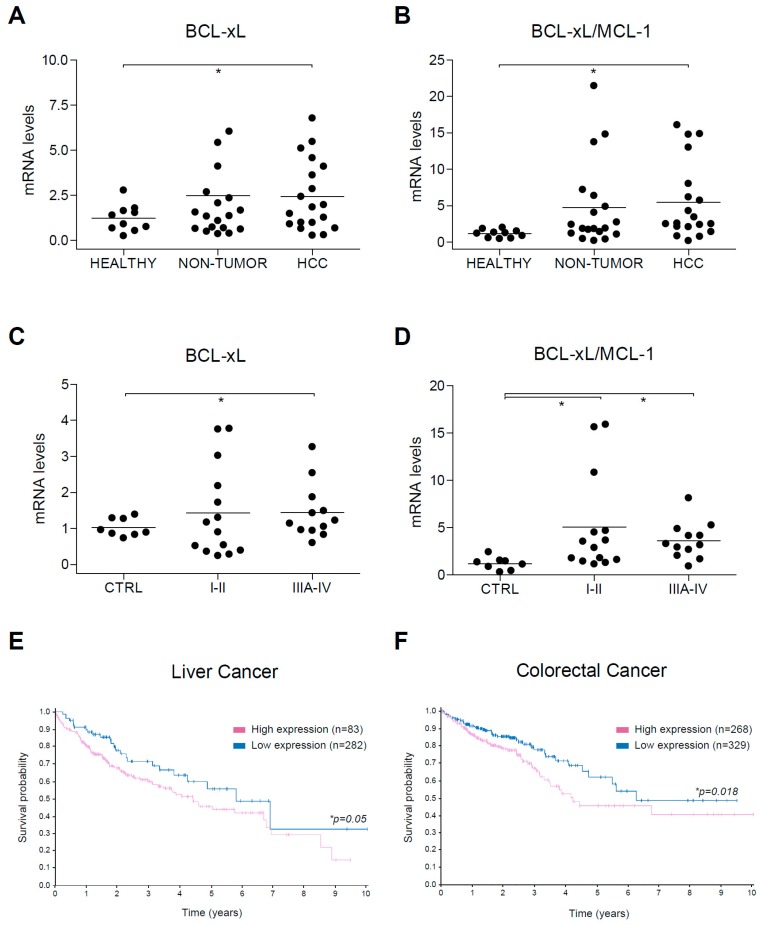
Alterations in BCL-xL mRNA levels and BCL-xL/MCL-1 ratio in HCC patients. (**A**) BCL-xL and (**B**) BCL-xL/MCL-1 mRNA levels were measured by qPCR in healthy liver (n = 10) and in cirrhotic and tumoral tissue from HCC patients (n = 12) with Hepatitis C virus (HCV) and/or Ethanol (EtOH etiology. * *p* < 0.05 vs. control. (**C**) BCL-xL and (**D**) BCL-xL/MCL-1 mRNA levels were measured by qPCR in a commercial mRNA array with healthy liver (n = 8) and tumoral tissue from HCC patients in different stages (I-II, n = 14; IIIA-IV, n = 12). (**E**,**F**) Representation of survival probability depending on BCL-xL expression (blue, high; purple, low) in patients with liver and colorectal cancer, respectively.

Since our results indicate that BCL-xL reduction by A-1331852 potentiates regorafenib activity and low MCL-1 levels expose A-1331852 anti-tumoral activity against HCC tumor cells, we focused our attention on BCL-xL and MCL-1 alterations exhibited by human HCC tumors. As previously observed in a set of human biopsies from control, HCC, and surrounding non-tumorous tissue [23], BCL-xL mRNA expression was increased in some HCC samples (Figure 8A), while MCL-1 reduction was general in all tumor tissues. As a consequence, the BCL-xL/MCL-1 ratio was significantly improved in the HCC tumor group (Figure 8B). 

To confirm these results, we used a commercial mRNA array with HCC tumors at different stages. Since our previously analyzed tumors were mostly small tumors (≤5 mm) in stage I-II, we wanted to compare this group with the one in stage III-IV. Once again, while BCL-xL upregulation was observed in specific tumors in both tumor groups (Figure 8C), the BCL-xL/MCL-1 ratio was significantly increased, both in stage I-II and in stage III-IV tumors (Figure 8D). These results suggest that BCL-xL upregulation is presented in HCC tumors and is frequently associated with a parallel MCL-1 reduction, a feature that could help A-1331852 anti-tumoral activity. Interestingly, no control sample exhibited a BCL-xL/MCL-1 ratio higher than 2.5, neither in our cohort (0/10) or in the commercial array (0/8). In contrast, a BCL-xL/MCL-1 ratio over 2.5 was detected in numerous tumors in our cohort (10/19) and the commercial array (18/26).

Finally, since our results suggest that BCL-xL upregulation could be detrimental for HCC treatment with regorafenib, but probably also for other treatments that generate mitochondrial sensitization, such as sorafenib, we checked in the Human Protein ATLAS data [37] if BCL-xL expression could be associated with a worse prognosis in liver cancer (Figure 8E). Interestingly, high BCL-xL mRNA levels were exhibited by many tumors (n=282) and have a worst 5-year survival prognostic (45%) than low BCL-xL levels (55%, n = 83, *p* = 0.05). 

Since regorafenib is also an FDA-approved drug for colorectal cancer treatment [38,39], and according to our data another potential candidate for treatment based on BCL-xL antagonism, we also analyzed BCL-xL levels in this tumor category (Figure 8F). The Atlas database analysis indicates that a BCL-xL increase in colorectal cancer is probably negative for patients having a worse 5-year survival prognosis (53%, n = 329) than low BCL-xL levels (71%, n = 268, *p* = 0.018). 

Consequently, liver and colorectal patients with increased BCL-xL tumor levels seem be associated with worse prognosis and may be candidates for a combination therapy with BCL-xL antagonists, such as A-1331852.

## 3. Discussion

Immunotherapy is a very promising field, but its application to HCC patients seems to be an option only for a low percentage of individuals [40]. Regorafenib, a multikinase inhibitor (MKI) with a broader inhibitory profile and greater pharmacological activity than sorafenib, has been approved as second-line therapy for advanced hepatocellular carcinoma (HCC) after sorafenib failure and for advanced colorectal cancer (CRC) and gastrointestinal stromal tumors (GISTs) after standard chemotherapy [38,39]. However, MKI therapy, despite being the best treatment for hepatocellular carcinoma, is still not very effective. Further improvement of MKI activity is important to detect among the intracellular mechanisms triggered by each drug those responsible for death induction. These altered pathways may allow identification of druggable targets for combination therapy or even for use as a single agent, if the drug is effective enough and markers for patient selection can be associated. In particular, when the mitochondrial functionality is compromised, the drug creates a tumor vulnerability that could be used to promote mitochondrial-dependent cell death [10,11,12,13]. Since mitochondria, through MOMP and the release of apoptogenic intermembrane proteins, can amplify the damage leading to cell death, it is important to identify the drugs that cause mitochondrial alteration and determine the molecular mechanism involved. 

In particular, if the BCL-2 network is affected, an interesting possibility arises since specific BH3-mimetics against BCL-2 proteins, such as BCL-2 (ABT-199), BCL-xL (A-1331852), or MCL-1 (A-1210477), have been designed and are tested in clinical trials. Therefore, chemotherapeutic agents that promote changes in BCL-2 proteins, once these modifications are characterized, become a probable target for combination therapy with BH3-mimetics. In fact, since the dependence on a BCL-2 protein is frequently related to its specific level, tumors with an elevated content of a specific BCL-2 family member can be treated in monotherapy with some BH3-mimetics, such as chronic and acute leukemia with ABT-199. However, what we expect to be more common is that BH3-mimetics could be administered in combination with standard chemotherapy, particularly in patients with high levels of the related protein. Of note, although the mRNA increases observed in HCCs from our cohort of patients and from the commercial array are significant, only specific individuals displayed very high levels of BCL-xL and the BCL-xL/MCL-1 ratio. It is tempting to speculate that these patients could particularly benefit from BCL-xL antagonism, mostly when, as observed in liver cancer and CRC patients, BCL-xL levels are inversely related to expected survival. Incidentally, when we separated groups depending on gender, females were much more sensitive to BCL-xL levels (*p* = 0.014, 5-year survival high 39% vs. 5-year survival low 61%). If this divergence is due to sex differences in the level of BCL-2 members, other apoptosis-related proteins in the liver, or the consequence of HCCs from different etiologies depending on each gender [41] should be further analyzed. 

A-1331852 is an orally bioavailable potent and selective BCL-xL inhibitor with a Ki value in the low nanomolar range, and affinity for other BCL-2 proteins, such as BCL-2 or MCL-1, of around 600 or 15,000 times less, respectively [29]. A-1331852 has been proposed as an agent in cancer therapy [42,43] and, more recently, as a senolytic compound [44]. Interestingly, through a dual mechanism acting on senescent cholangiocytes and activated fibroblasts, A-1331852 ameliorates liver fibrosis in mice [45]. Therefore, BCL-xL inhibition, besides a direct effect on HCC survival, may change the protumoral microenvironment in which HCC develops by eliminating hepatic senescent cells and activated fibroblasts. In this sense, experiments using liver spheroids combining liver cancer cells and activated hepatic stellate cells could be an interesting in vitro model to study this additional effect of BCL-xL inhibition. In fact, our preliminary results indicate that A-1331852 efficiently reduces tumor growth in HepG2/LX2 spheroids alone and particularly in combination with regorafenib, and in vivo mice experiments are ongoing. Moreover, other effects of BCL-xL reduction should not be discarded, since A-1331852 affects other genes important in HCC biology. The recent discovery of PUMA controlling the metabolic switch in HCC via direct interaction with the mitochondrial pyruvate carrier suggests that other actions of BCL-2 proteins could be expected [46]. 

Other BH3-mimetics, such as ABT-199 (venetoclax, BCL-2 inhibitor), FDA approved for chronic and acute leukemia, or ABT-263 (navitoclax, BCL-2 and BCL-xL inhibitor), are in clinical trials despite their associated hematological side effects. In particular, platelet survival is dependent on BCL-xL expression, and thrombocytopenia could be presented after administration of BCL-xL inhibitors, as observed in navitoclax studies. In addition, navitoclax-induced BCL-2 inhibition may also reduce the neutrophil count, at least in combination with other therapies [29]. In this sense, BCL-xL-selective inhibitors, such as A-1331852, will avoid dose-limiting neutropenia although its platelet effect may complicate its use as a single agent, particularly in some cirrhotic patients with HCC. However, as observed in combination with regorafenib, A-1331852 can be effective at very low concentrations, most probably before thrombocytopenia became dose limiting. In agreement, navitoclax’s effect on the platelet count can be attenuated by careful dosing, as observed in clinical trials in patients with lymphoid malignancies [47]. 

Interestingly, a BCL-xL proteolysis-targeting chimera (PROTAC), which targets BCL-xL to the Von Hippel-Lindau (VHL) E3 ligase for degradation, has recently been designed [48]. This selective BCL-xL PROTAC degrader exhibits safe and potent antitumor activity but considerably less toxicity to platelets than ABT-263, since VHL is poorly expressed in platelets. These novel data illustrate the importance of BCL-xL in specific tumors and the possibility to circumvent the side effects related to BCL-xL deficiency in particular cells. 

In summary, our data support the concept that BH3-mimetics are remarkable compounds to combine with cancer therapy when the BCL-2 network is altered. In this sense, regorafenib perturbation of the BCL-2 family creates a mitochondrial vulnerability that A-1331852 can exploit. Through MOMP and caspase activation, BCL-xL inhibition potentiates regorafenib action in in vitro and in vivo HCC models. Therefore, A-1331852 or other strategies directed to eliminate BCL-xL, such as PROTACS, should be contemplated as potential candidates for combination therapy with regorafenib in HCC treatment and probably in other cancers that exhibit BCL-xL overexpression. 

## 4. Materials and Methods 

### 4.1. Reagents

Dulbecco’s modified eagle’s medium (DMEM), trypsin-EDTA, penicillin-streptomycin and dimethyl sulfoxide (DMSO), MTT (3-(4,5-dimethylthiazol-2-yl)-2,5-diphenyl tetrazolium bromide) (M2128), Hoechst 33258 (B1155), Crystal Violet (C0755), and DCF (D6883) were purchased from Sigma-Aldrich (St. Louis, MO, USA). All tissue culture-ware was from Nunc (Roskilde, Denmark). Biotin Blocking System, peroxidase substrate (DAB), and peroxidase buffer were from DAKO (Glostrup, Denmark). Proteinase inhibitors were from Roche (Madrid, Spain). ECL Western blotting substrate was from Pierce (Thermo Fisher Scientific, Rockford, IL, USA). BCL-2 siRNA (h) (sc-29214), BCL-xL siRNA (h) (sc-43630), and scrambled controls were purchased from Santa Cruz Biotechnology (Dallas, TX, USA), while a second siRNA for BCL-2 (ID#s1915) and for BCL-xL (ID#s1920) were obtained from Ambion Life technologies (Carlsbad, CA, USA). Lipofectamine2000 (11668-027), Novex Sharp Pre-stained Protein Standard (LC5800), and JC-1 (T-3168) were from Invitrogen Life Technologies (Carlsbad, CA, USA). Sorafenib (BAY 43-9006, Nexavar) and Regorafenib (BAY 73-4506, Stivarga) are manufactured by Bayer. A-1331852 and ABT-199 (Venetoclax) were purchased from Selleckchem (Houston, TX, USA). 

### 4.2. Cell Culture and Biochemical Analysis

Human liver tumor cell lines Hep3B, PLC/PRF/5 and HepG2 (European Collection of Animal Cell Cultures (ECACC)), and human hepatic stellate cell line LX2 [49] were grown in DMEM (10% FBS) at 37 °C and 5% CO_2_. Regorafenib-resistant hepatoma cells were maintained at 2 μM and kept without drug at least one week before experiments. Primary mouse hepatocytes were obtained after collagenase digestion [50] and cultured on collagen-coated plates one day before analysis. 

### 4.3. MTT Assay

Cell viability was determined by MTT (3-(4,5-dimethylthiazol-2-yl)-2,5-diphenyl tetrazolium bromide) assay. In total, 1 × 10^4^ cells/well were seeded in a 96-well plate and incubated at 37 °C and 5% CO_2_. Cells were treated with regorafenib and A-1331852, ABT-199, and A-1210477 for 16–24 h before 10 µL of MTT reagent (5 mg/mL) addition and incubation for 2 h. Formazan crystals were dissolved with 100 µL of 1-propanol. Absorbance was measured in a plate reader (Multiskan® Spectrum, Thermo Fisher Scientific, Rockford, IL, USA) at 570 and 630 nm. 

### 4.4. Crystal Violet Staining

First, 8 × 10^4^ cells were seeded into 12-well plates and kept at 37 °C in 5% CO_2_. Cells were treated and left for three days until they were fixed with 10% formalin for 5 min. Crystal violet was added for 30 min and after that they were washed twice with water. Plates were drained and photos were taken.

### 4.5. Caspase-3 Activity Assay

First, 3 × 10^4^ cells were seeded in a 12-well plate. After treatments, cells were scrapped with 50 mM Hepes (pH 7.4), 5 mM CHAPS, and DTT 5mM. For Caspase-3 activity, 50 μg of protein extraction were added in 200 uL of assay buffer containing 20 mM Hepes, 5% sucrose, 0.1% CHAPS, 2 mM EDTA, and 5 mM DTT, pH 7.4, and 50 μM of the substrate Ac-DEVD-AFC (Santa Cruz Biotechnology). Detection of AFC after substrate cleavage was recorded at time intervals of 15 min, at emission 505 nm, and excitation at 400 nm. A unit of caspase-3 activity is the amount of active enzyme necessary to produce an increase in 1 fluorescence unit in Spectramax Gemini XS fluorimeter. Results are usually represented as an arbitrary unit/h/μg protein.

### 4.6. Hoechst Staining

Cells were seeded at 5 × 10^4^ cells/well in 12-well plates, treated for 8 h. Hoechst 33258 was added at 1/1000 and incubated for 30 min. After being washed, images were taken using Olympus IX-70 microscope with the CC-12 FW camera. Photos of 12 random fields were taken. Condensed nuclei were counted with ImageJ software. 

### 4.7. Mitochondrial Membrane Potential Assay

JC-1 is a fluorescent cationic dye (C5,5′,6,6′-tetrachloro-1,1′,3,3′-tetraethylbenzimidazolyl- carbocyanine iodide) used as an indicator of mitochondrial potential in cells. Mitochondrial depolarization is assessed by a decrease in the red (J-aggregates)/green (J-monomers) fluorescence intensity ratio. To determine the mitochondrial membrane potential, 1 × 10^4^ cells/well were seeded in 96-well plates and incubated at 37 °C and 5% CO_2_. Cells were treated, and after, JC-1 dye was incubated for 15 min. DMSO (0.05%) was used as the control. Photos were taken with a Leica-CTR4000 microscope and LAS software.

### 4.8. 3D Tumor Liver Spheroids Generation

Cellular spheroids were generated and plated in 96-well plates with a bottom coat of agarose [51,52]. Tumor liver spheroids were kept at 37 °C and 5% CO_2_ for seven days and spheroid growth monitored daily.

### 4.9. Immunoblot Analysis

Cell lysates were prepared in RIPA buffer plus proteinase inhibitors. Samples containing 10 to 30 µg were separated by 10%–15% SDS-PAGE. Proteins were transferred to nitrocellulose membranes, blocked in 5% nonfat milk for 1 h at room temperature, and incubated overnight at 4 °C with the primary antibodies: MCL-1 (S-19, sc-819) 1:250 rabbit; BCL-2 (C-2, sc-7382) 1:250 mouse; BCL-xL (H-5, sc-8392) 1:250 mouse; PARP-1 (H-250, sc-7150) 1:250 rabbit; BIM (H-191, sc-11425) 1:250 rabbit; BAX (N-20, sc-493) 1:1000 rabbit; TOM20 (sc-11415) 1:500 rabbit; BAK (AT38E2, sc-517390) 1:250 mouse; Cytochrome C (sc-1356) 1:250 mouse were from Santa Cruz Biotechnology; Cleaved Caspase-3 (D175, #9661S) 1:1000 rabbit from Cell Signaling, and β-Actin (A3854) 1:40,000 conjugated to HRP from Sigma-Aldrich.

### 4.10. RNA Isolation and Real Time RT-PCR

Total RNA was isolated with TRIzol reagent. 1µg of RNA was reverse transcribed with AN iScript™ cDNA Synthesis Kit (Biorad, Berkeley, CA, USA) and real-time PCR was performed with iTaq™ Universal SYBR^®^ Green Supermix (Biorad) following the manufacturer’s instructions. The primers sequences used were:

human BCL-2: Fw 5′-GGAGGATTGTGGCCTTCTTT-3′; Rv 5′-GCCGTACAGTTCCACAAAGG-3′ human BCL-xL: Fw 5′-GGATGGCCACTTACCTGA-3′; Rv 5′-CGGTTGAAGCGTTCCTG-3′
human MCL-1: Fw 5′-ATGCTTCGGAAACTGGACAT-3′; Rv 5′-TCCTGATGCCACCTTCTAGG-3′ human ′-Act: Fw 5′-AGAAAATCTGGCACCACACC-3′ Rv 5′-AGAGGCGTACAGGGATAGCA-3′

### 4.11. Immunohistochemical Staining

Livers were fixed and paraffin embedded. Sections were routinely stained with Hematoxylin&Eosin (7-μm) or incubated with mAb anti-PCNA antibody (PC10) (1:200 dilution, sc-56, Santa Cruz Biotechnology) as previously indicated [53]. The slices were examined with a Zeiss Axioplan microscope equipped with a Nikon DXM1200F digital camera. The PCNA cell count was quantified in four randomly selected fields from each animal and analyzed using ImageJ software. Ki-67 staining was performed using a specific antibody (sc-23900, 1:200 mouse) from Santa Cruz Biotechnology.

### 4.12. Tumor Animal Models

All animal procedures were performed according to protocols approved by the Animal Experimentation Ethics Committee from the University of Barcelona (ethic code: #9850). For the subcutaneous tumor model, male Swiss nude mice, 5–6 weeks old, were kept under pathogen-free conditions with free access to standard food and water. HepG2 sorafenib-resistant cells (5 × 10^6^) or BCLC9 cells (2.5 × 10^6^) were injected subcutaneously into the flanks of mice in 100 μL DMEM without FBS, as previously reported [19,23,53]. Treatments with A-1331852 (25 mg/Kg body weight), regorafenib (30 mg/Kg), or vehicle (12.5% Cremophor, 12.5% ethanol, 75% sterile saline) were delivered daily via oral gavage. Tumors were measured periodically with a Vernier caliper, and the volume was calculated as length × width^2^ × 0.5. 

### 4.13. Gene Array

Predesigned 384-well human Liver cancer panel (SAB Target List, H384 Cat#10034526) and Cell Death (SAB Target List, H384 Cat#10034460) for SYBR Green detection (Bio-rad) were used following the manufacturer’s instructions, as previously reported [54].

### 4.14. cDNA Array

TissueScan™ cDNA Array (Liver Cancer cDNA Array I, Origene) was used to quantify BCL-xL and MCL-1 levels in tumor and normal tissues. Tissue cDNAs of each array are synthesized from high quality total RNAs of pathologist verified tissues, normalized and validated with β-actin in two sequential qPCR analyses, and provided with clinical information and QC data. Our array contained cDNA from 48 samples covering 8-normal, 7-Stage I, 8-II, 8-IIIA, and 3-IV in identical plates (LVRT101). BCL-xL and MCL-1 levels were calculated by qPCR as previously indicated.

### 4.15. HCC Patient Study and ATLAS Database Information

Tumor and cirrhotic tissue from 19 patients diagnosed with HCC and treated at the Clinic Hospital in Barcelona, and 10 healthy liver samples from patients subjected to surgery due to colorectal cancer without any diagnosed liver disease, were included [23]. Patient data is included in Supplementary Table S1. Patients gave informed consent according to the principles embodied in the Declaration of Helsinki. 

Data showing survival probability depending on the level of expression of BCL-xL were retrieved from: https://www.proteinatlas.org/ENSG00000171552-BCL2L1/pathology/liver+cancer for liver cancer; https://www.proteinatlas.org/ENSG00000171552-BCL2L1/pathology/colorectal+cancer for colorectal cancer.

### 4.16. Statistical Analyses

Results are expressed as mean ± standard deviation and n = 3, unless indicated. Statistical comparisons were usually performed using unpaired 2-tailed Student’s t test, and 1-way ANOVA followed by Newman–Keuls multiple comparison test (GraphPad Prism) was used for data quantification from patients. A *p* value less than 0.05 was considered significant. 

## 5. Conclusions

In HCC models, regorafenib induces changes in BCL-2 family proteins, priming mitochondrial cell death induced by BH3-mimetics, and allowing the BCL-xL inhibitor A-1331852 to enhance regorafenib efficacy. 

BCL-xL increase, associated with a poor prognosis in liver and colorectal cancer, could be an interesting molecular marker for regorafenib/A-1331852 combinatory treatment in HCC patients.

## Supplementary Materials

The following are available online at https://www.mdpi.com/2072-6694/12/2/332/s1, Table S1: Data from the patients included in the study, Figure S1, Caspase-3 activity in HepG2 cells treated with regorafenib and A-1331852, Figure S2, mRNA levels of MCL-1 in HepG2 cells after sorafenib treatment, Figure S3, Ki-67 expression in regorafenib/A-1331852-treated BCLC9 tumors, Figure S4, Cell viability of primary mouse hepatocytes after A-1331852 exposure, Figure S5, Cell viability of human hepatic stellate cells LX2 after A-1331852 exposure, Figure S6, TUNEL staining in regorafenib+A-1331852-treated BCLC9 tumors, The whole western blot figures.
