# Supplementary material for: Regorafenib Alteration of the BCL-xL/MCL-1 Ratio Provides a Therapeutic Opportunity for BH3-Mimetics in Hepatocellular Carcinoma Models
Source: Cancers (Basel). 2020 Feb 1;12(2):332. doi: 10.3390/cancers12020332 (PMC7073154; doi:10.3390/cancers12020332)
Supplement: Supplementary file 1 [file cancers-12-00332-s001.pdf]

# Supplementary materials: Regorafenib alteration of the BCL-xL/MCL-1 ratio provides a therapeutic opportunity for BH3-mimetics in hepatocellular carcinoma models

**Blanca Cucarull, Anna Tutusaus, Miguel Subías, Milica Stefanovic, Tania Hernáez, Loreto Boix, María Reig, Pablo García de Frutos, Montserrat Marí, Anna Colell, Jordi Bruix,** and **Albert Morales**

**Table S1.** Patient's data.

| GENDER | AGE | Underlying liver disease | ETHIOLOGY | Virus hepatitis | AFP (ngr/mL) | TUMOR SIZE (cm) | Tumor | Vascular Invasion |
|---|---|---|---|---|---|---|---|---|
| M | 67 | cirrhosis | alcohol | NO | | 4,5 | single | NA |
| M | 54 | cirrhosis | VHC+VHB+alcohol | VHC+VHB | 15 | 1,3 | single | NA |
| M | 50 | cirrhosis | VHC+alcohol | VHC | 19 | 1,4 | single | NA |
| M | 61 | chronic hepatitis | VHC | VHC | 20 | 1,8 | single | Yes |
| M | 33 | chronic hepatitis | VHB | VHB | 4 | 1,1 | single | No |
| F | 61 | cirrhosis | VHC | VHC | 11 | 2,1 | single | No |
| M | 56 | cirrhosis | VHC | VHC | 2 | 5,2 (principal) | multinodular | No |
| M | 68 | cirrhosis | alcohol | No | 1515 | 5 | single | Yes, microvascular |
| M | 61 | cirrhosis | alcohol | No | 2 | 3,5 | single+1 satelite | No |
| M | 47 | cirrhosis | VHC | VHC | 6 | 2,5 | single | No |
| M | 75 | cirrhosis | VHC | VHC | 23 | 1,1 ( principal) | binodular | No |
| F | 69 | cirrhosis | VHC | VHC | 7 | 1,2 | single | Yes, microvascular |
| M | 78 | cirrhosis | VHC | VHC | | 4,5 | single | No |
| M | 73 | cirrhosis | VHC | VHC | 2 | 2,1 | single | No |
| M | 67 | cirrhosis | VHC | VHC | 22 | 2,1 | single | No |
| M | 63 | cirrhosis | VHC | VHC | 2 | 2,2 | single | No |
| M | 63 | chronic hepatitis | VHC | VHC | 9 | 6 | single | No |
| M | 48 | cirrhosis | VHC | VHC | 7 | 7,5 | single | Yes, peritumoral |
| M | 64 | cirrhosis | alcohol | NO | 6 | 4,5 | single | No |
| M | 68 | healthy liver | NA | NO | NA | NA | NA | NA |
| F | 70 | healthy liver | NA | NO | NA | NA | NA | NA |
| F | 47 | healthy liver | NA | NO | NA | NA | NA | NA |
| F | 50 | healthy liver | NA | NO | NA | NA | NA | NA |
| F | 55 | healthy liver | NA | NO | NA | NA | NA | NA |
| M | 36 | healthy liver | NA | NO | NA | NA | NA | NA |
| M | 36 | healthy liver | NA | NO | NA | NA | NA | NA |
| M | 55 | healthy liver | NA | NO | NA | NA | NA | NA |
| M | 37 | healthy liver | NA | NO | NA | NA | NA | NA |
| F | 73 | healthy liver | NA | NO | NA | NA | NA | NA |

# Supplemental Figure 1

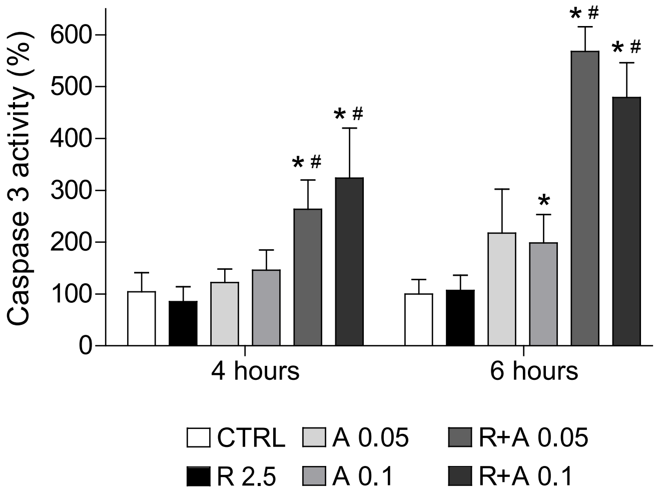


HepG2 cells were exposed to regorafenib (R, 2.5 µM) with or without A-1331852 (A, 0.1-0.05 µM) and caspase-3 activity was determined fluorimetricaly at four and six hours. *P< 0.05 vs. control cells, #P< 0.05 vs. regorafenib-treated cells. (n=3)

# Supplemental Figure 2

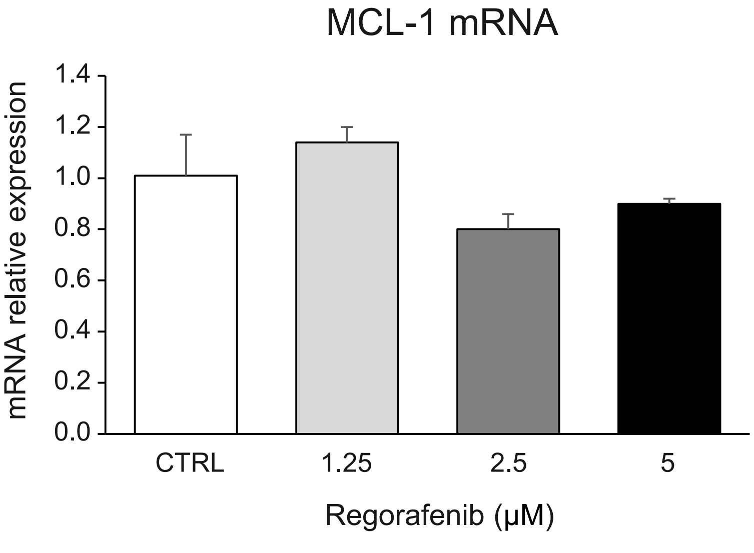


HepG2 cells were exposed to regorafenib (R, 1-5 µM) for 16 h and mRNA levels of MCL-1 were determined by qPCR. (n=2)

# Supplemental Figure 3

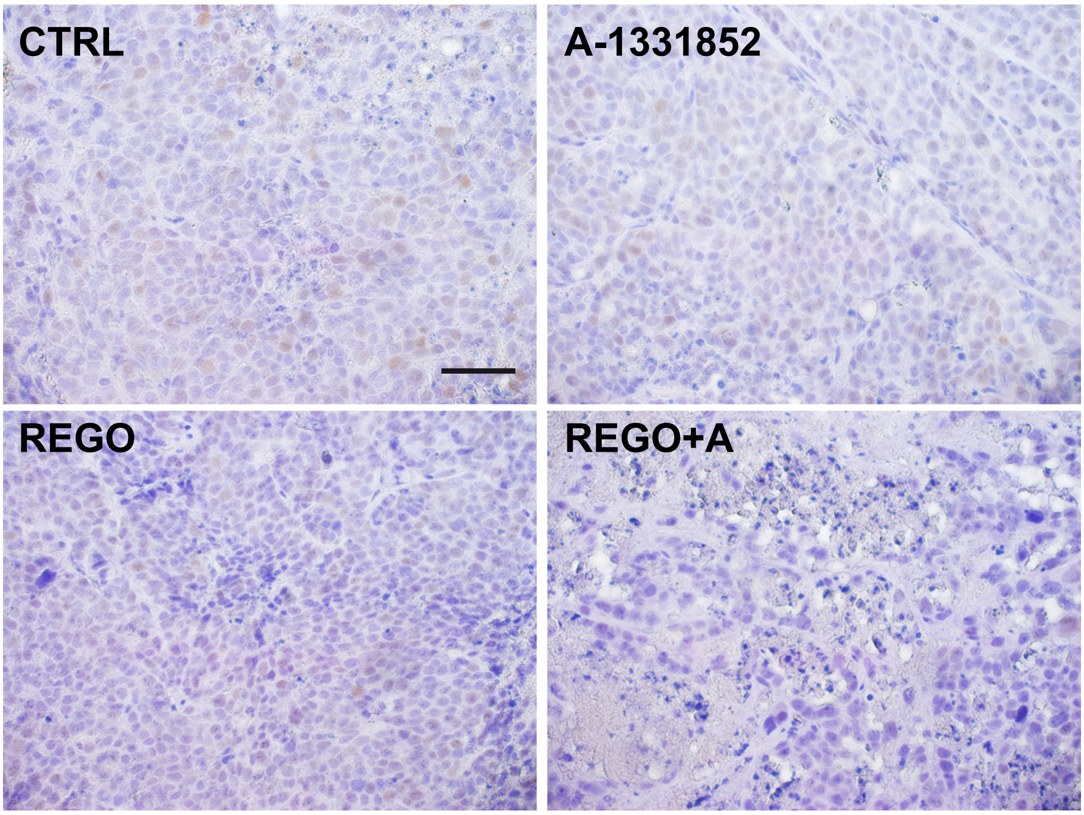


Representative images of Ki-67 expression in tumor samples from BCLC9 PDXs (scale bar, 50 μm).

# Supplemental Figure 4

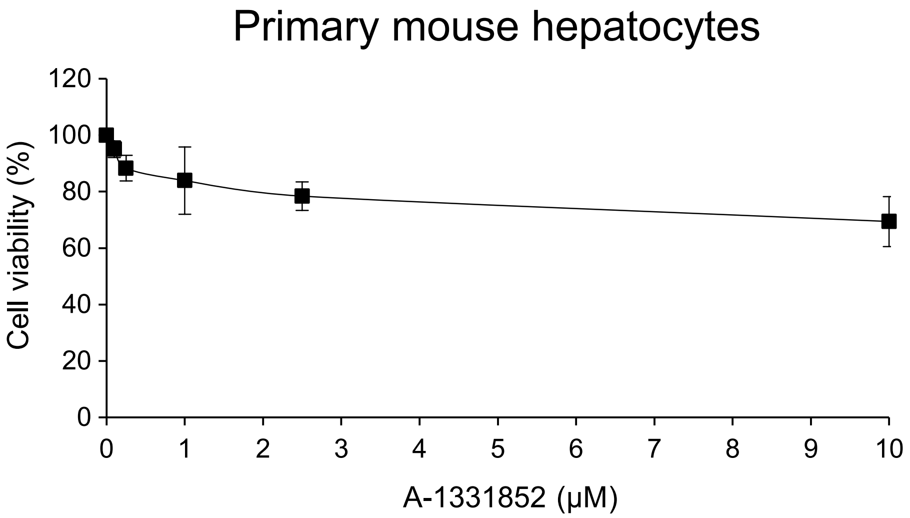


Primary mouse hepatocytes cultured on collagen plates were exposed to A-1331852 at different concentrations (up to 10 μM) and MTT assays performed 16 h. (n=4)

# Supplemental Figure 5

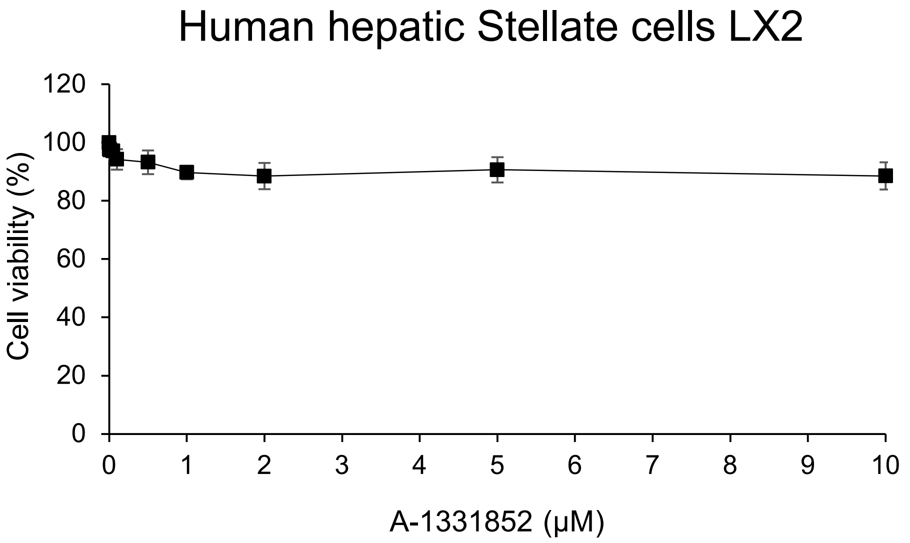


Human hepatic Stellate cells LX2 were exposed to A-1331852 at different concentrations (up to 10 µM) and MTT assays performed 16 h. (n=4)

# Supplemental Figure 6

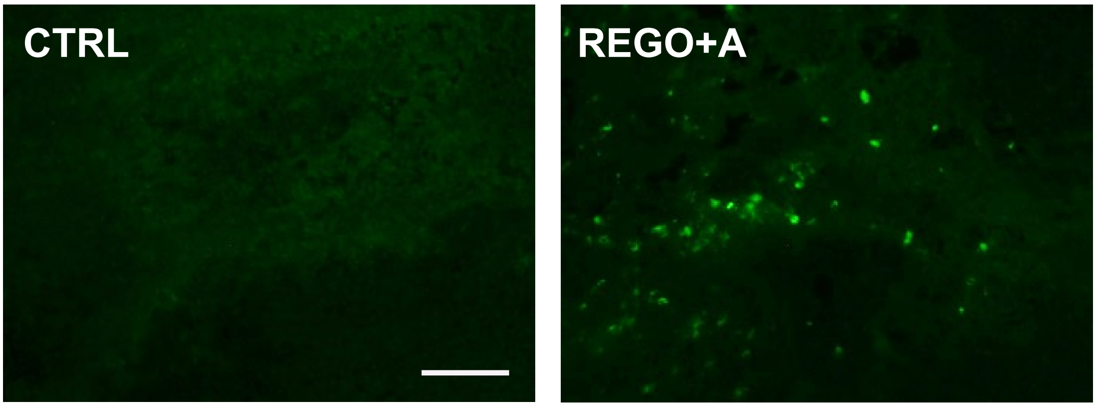


Representative images of TUNEL staining in tumor samples from R HEPG2 CDXs (scale bar, 100 µm).

The whole western blot

**Figure 2F**

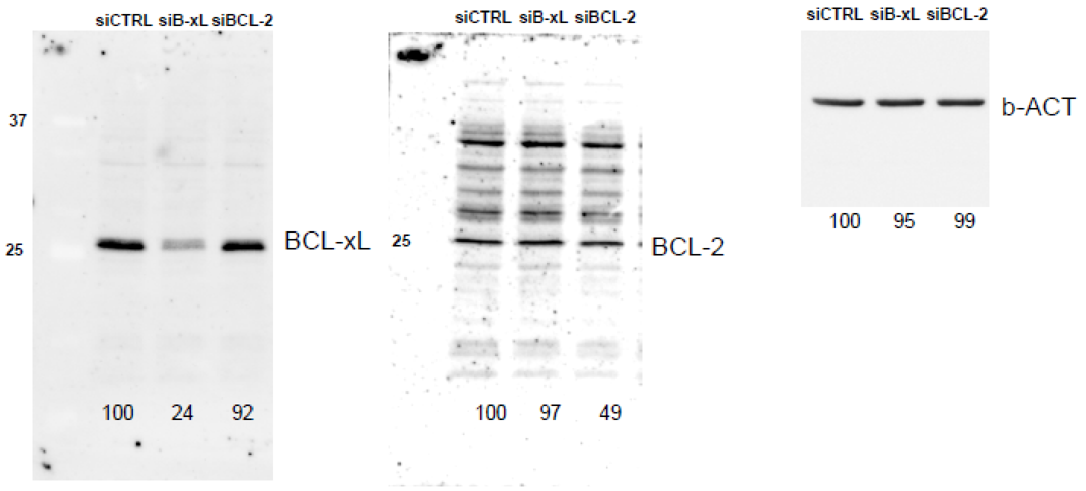


**Figure 4B**

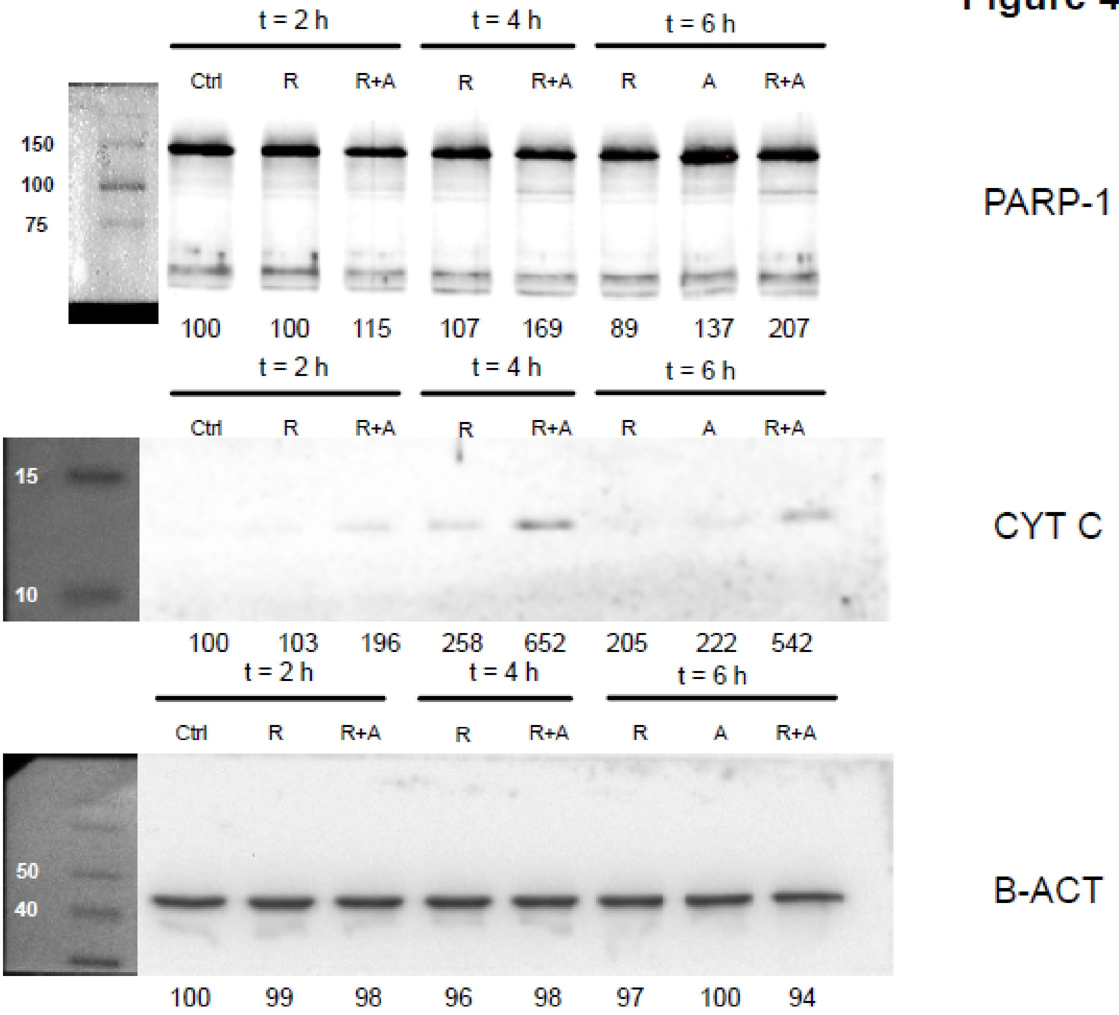

Figure 5A

HEP3B

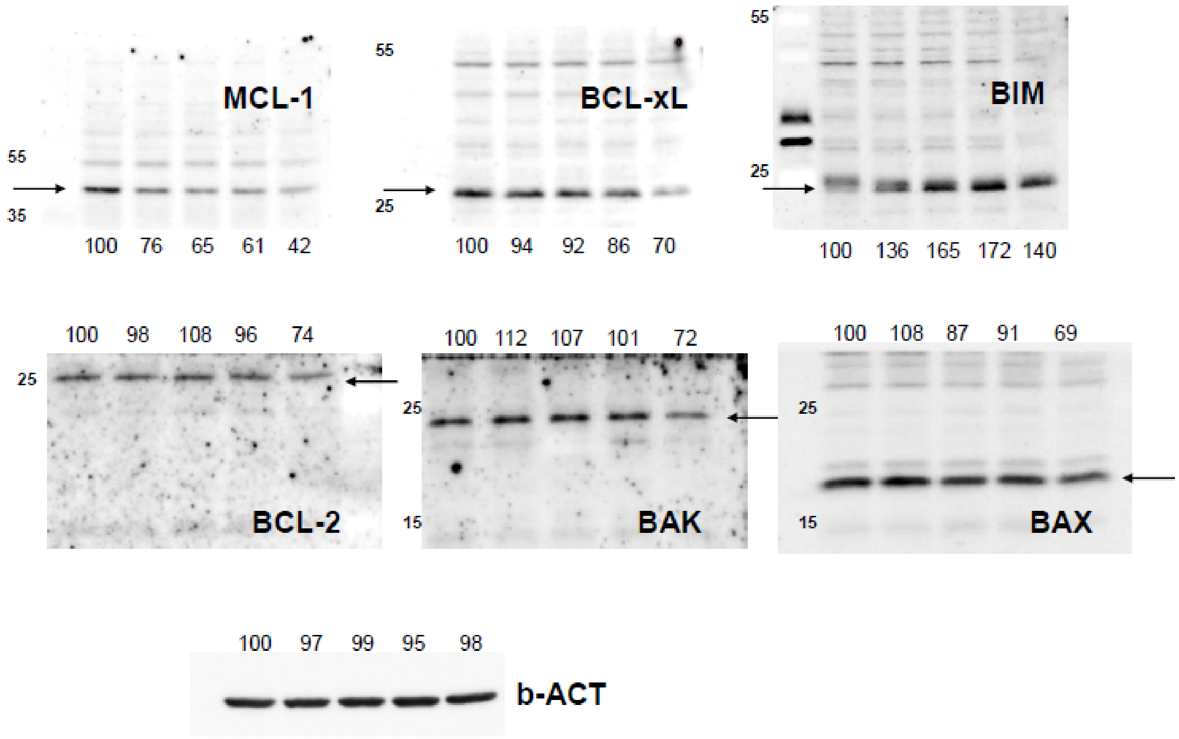


Figure 5A

HEPG2

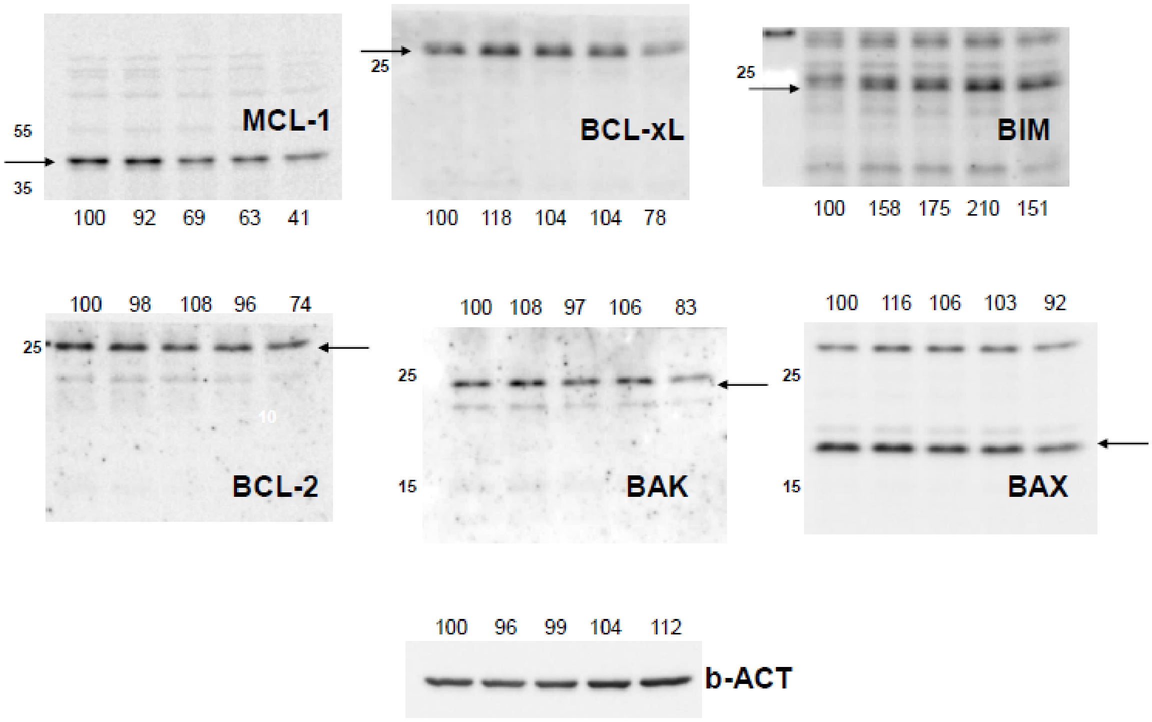

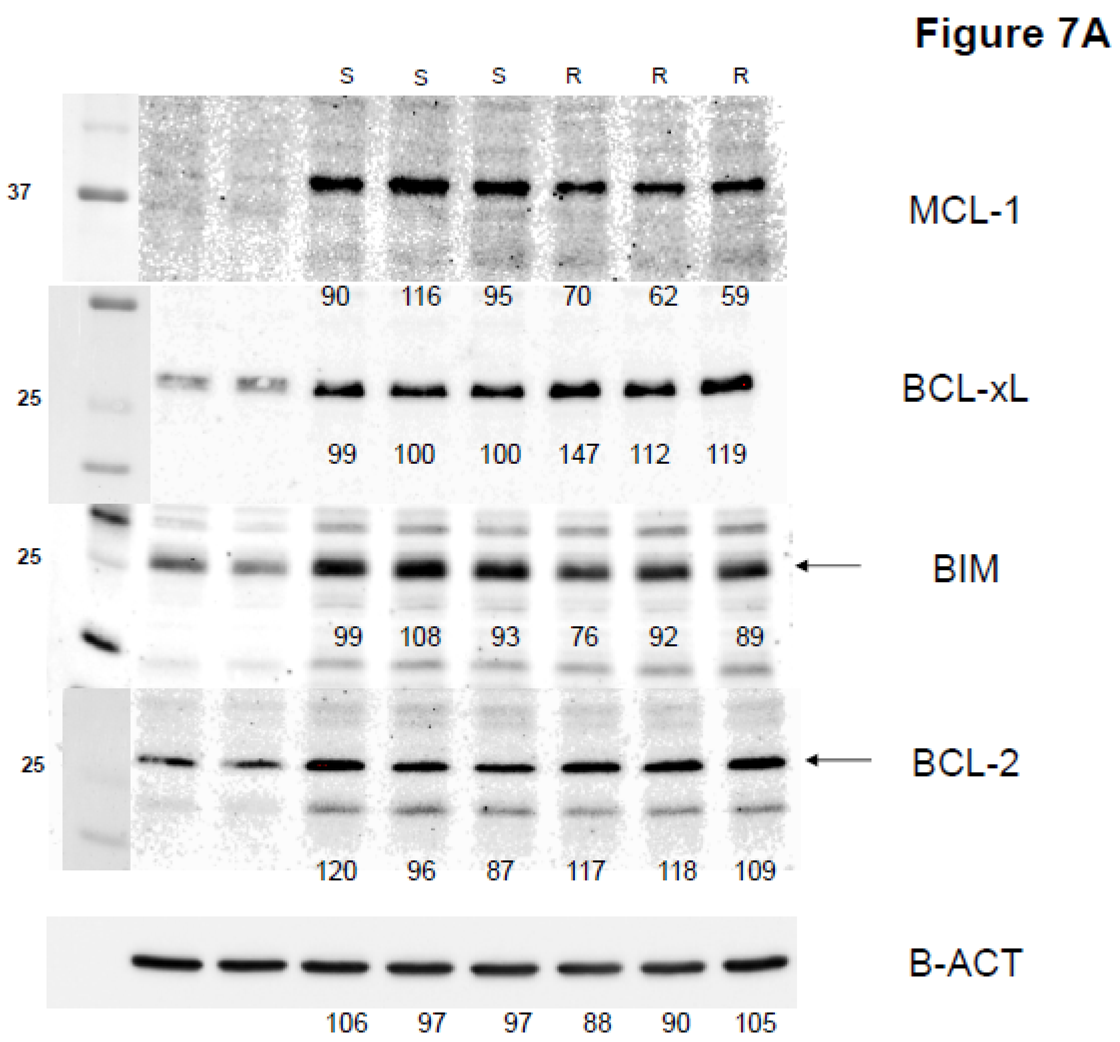
Figure 7A
S S S R R R
37
MCL-1
90 116 95 70 62 59
25
BCL-xL
99 100 100 147 112 119
25
BIM
99 108 93 76 92 89
25
BCL-2
120 96 87 117 118 109
B-ACT
106 97 97 88 90 105

Figure 4B

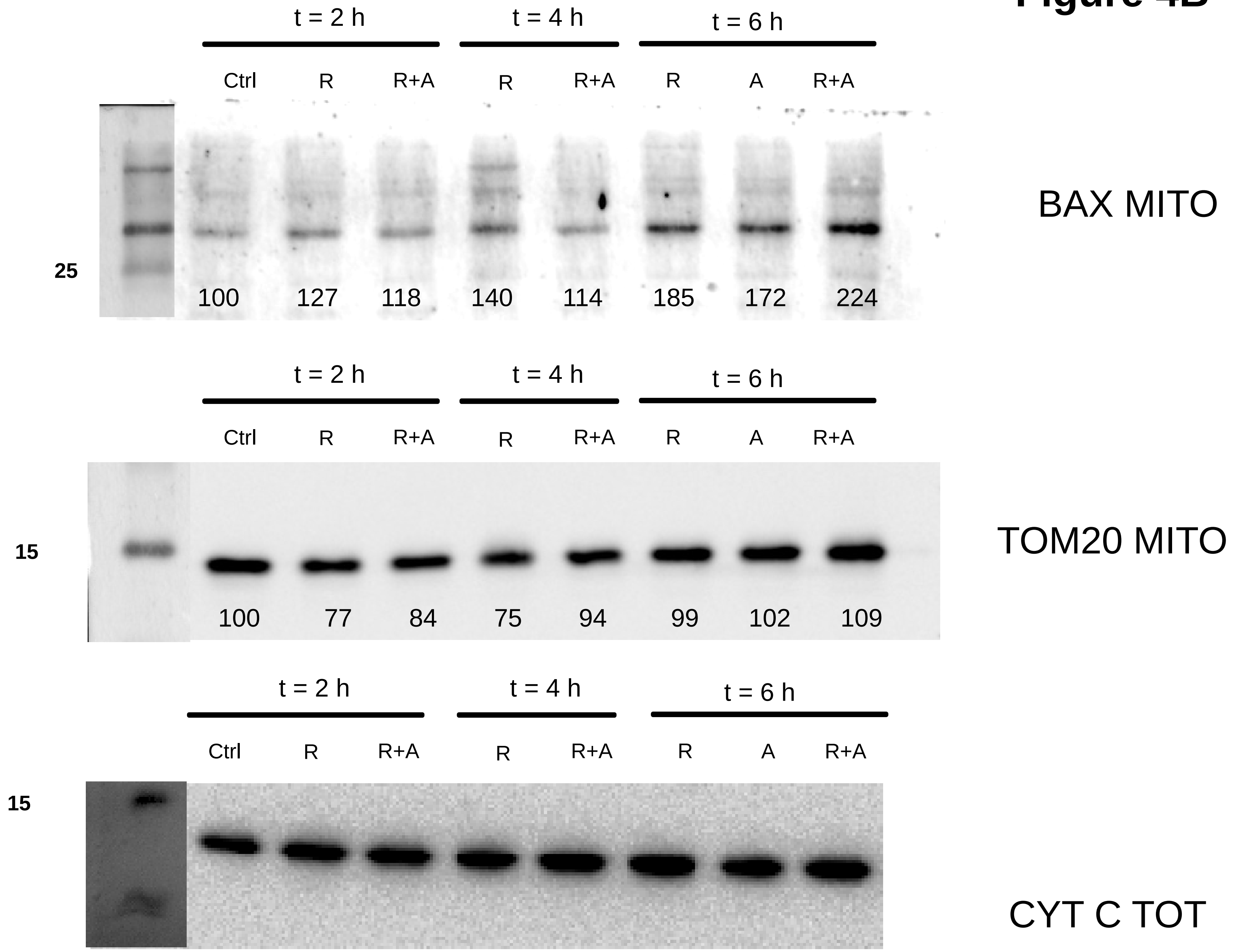
t = 2 h
t = 4 h
t = 6 h
Ctrl
R
R+A
R
R+A
R
A
R+A
25
100
127
118
140
114
185
172
224
BAX MITO
t = 2 h
t = 4 h
t = 6 h
Ctrl
R
R+A
R
R+A
R
A
R+A
15
100
77
84
75
94
99
102
109
TOM20 MITO
t = 2 h
t = 4 h
t = 6 h
Ctrl
R
R+A
R
R+A
R
A
R+A
15
CYT C TOT

# Figure 4B

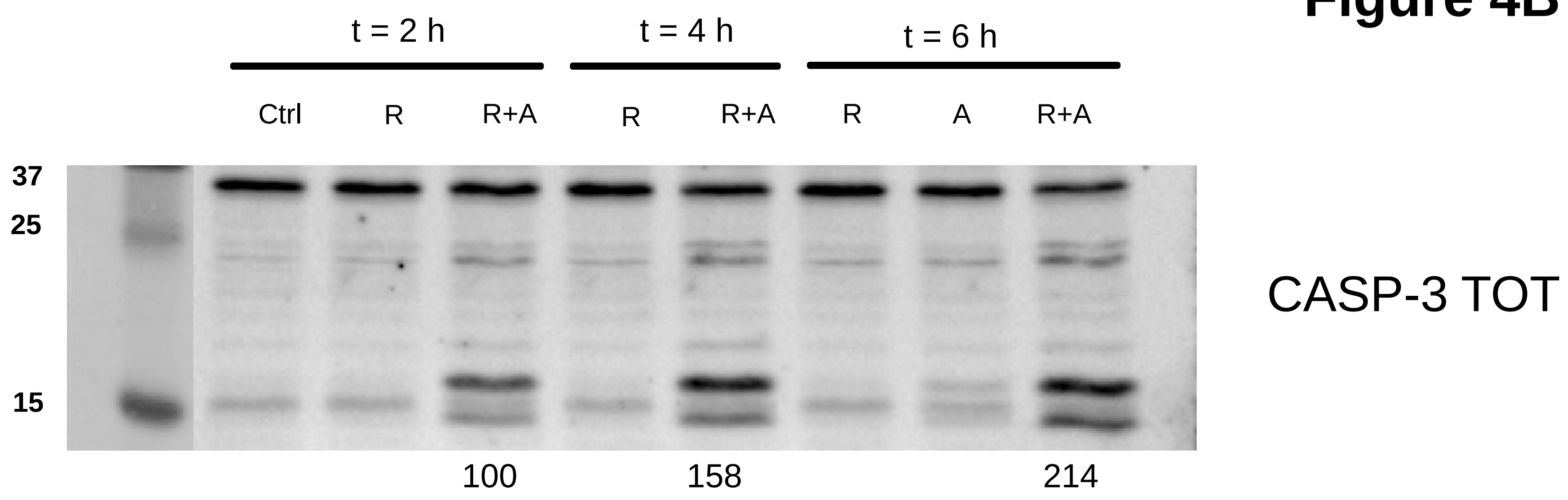

# Figure 4C

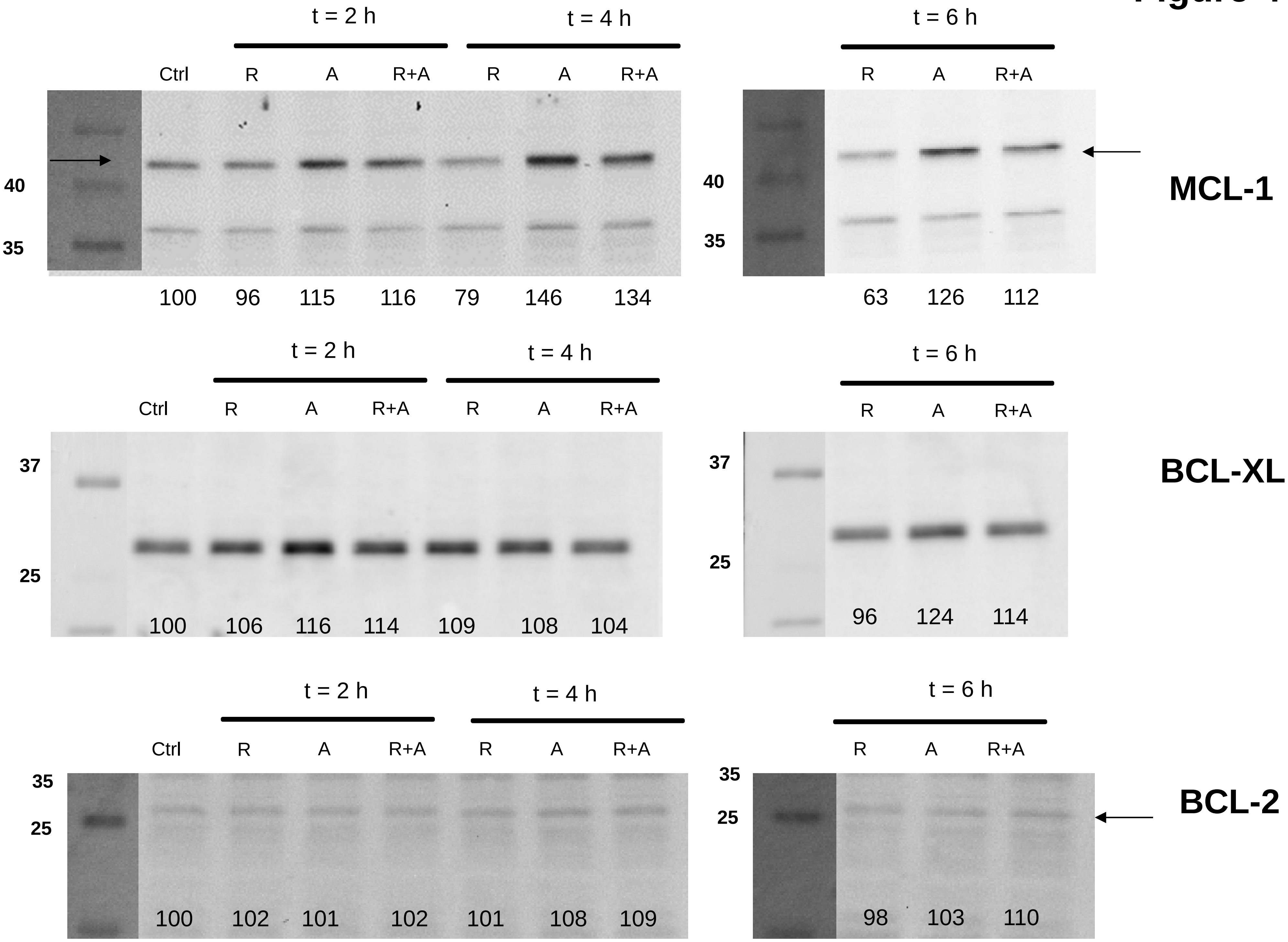

t = 2 h
t = 4 h
t = 6 h
Ctrl R A R+A R A R+A
R A R+A
40
35
100 96 115 116 79 146 134
63 126 112
MCL-1
t = 2 h
t = 4 h
t = 6 h
Ctrl R A R+A R A R+A
R A R+A
37
25
100 106 116 114 109 108 104
96 124 114
BCL-XL
t = 2 h
t = 4 h
t = 6 h
Ctrl R A R+A R A R+A
R A R+A
35
25
100 102 101 102 101 108 109
98 103 110
BCL-2

# Figure 4C

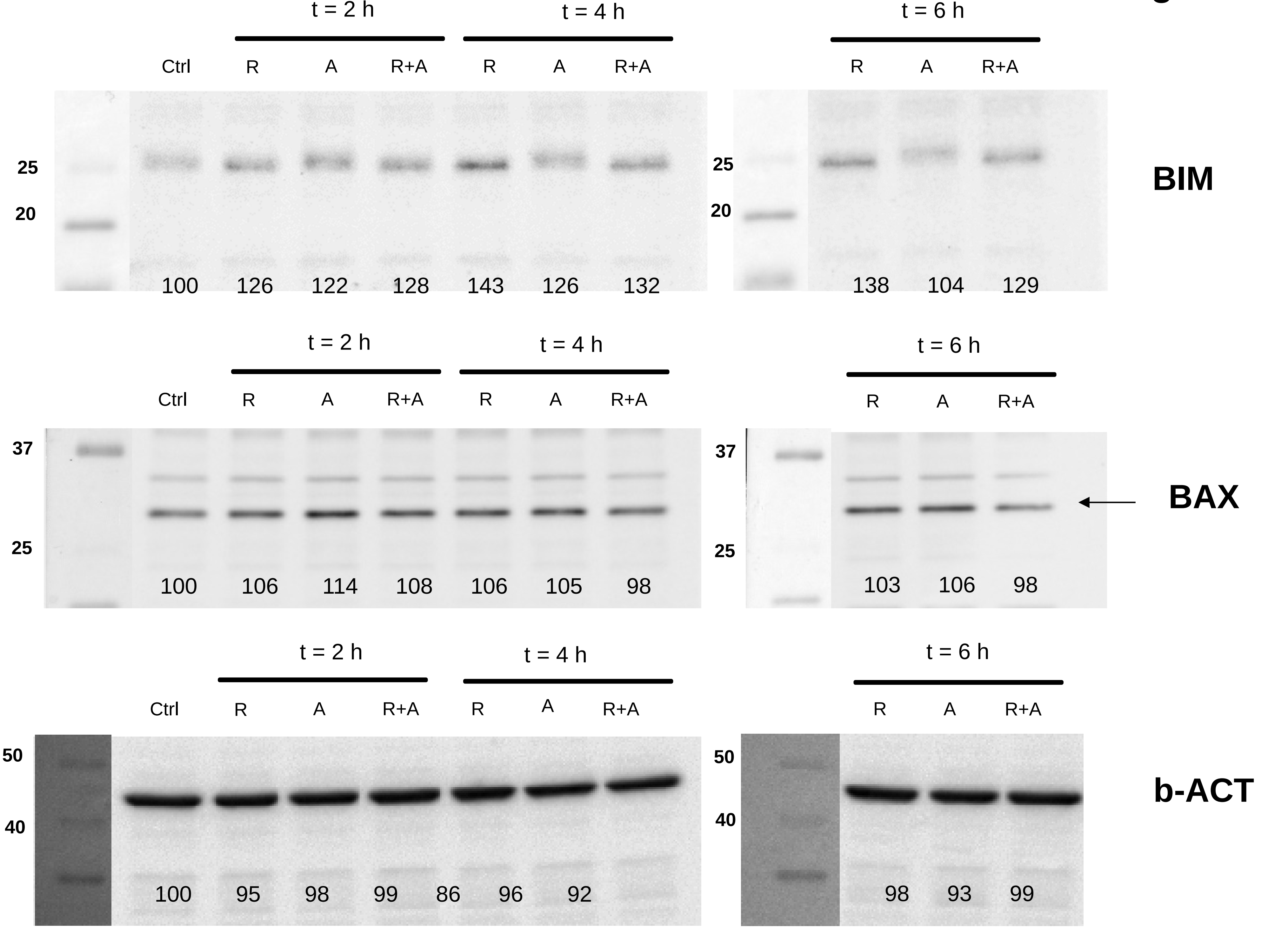
t = 2 h
t = 4 h
t = 6 h
Ctrl R A R+A R A R+A
R A R+A
25
20
BIM
100 126 122 128 143 126 132
138 104 129
t = 2 h
t = 4 h
t = 6 h
Ctrl R A R+A R A R+A
R A R+A
37
25
BAX
100 106 114 108 106 105 98
103 106 98
t = 2 h
t = 4 h
t = 6 h
Ctrl R A R+A R A R+A
R A R+A
50
40
b-ACT
100 95 98 99 86 96 92
98 93 99
